# The structure of Brazilian Amazonian gut microbiomes in the process of urbanisation

**DOI:** 10.1038/s41522-021-00237-0

**Published:** 2021-08-05

**Authors:** Ana Paula Schaan, Dionison Sarquis, Giovanna C. Cavalcante, Leandro Magalhães, Eliene R. P. Sacuena, John Costa, Dennyson Fonseca, Vanessa J. Mello, João F. Guerreiro, Ândrea Ribeiro-dos-Santos

**Affiliations:** 1 Programa de Laboratório de Genética Humana e Médica, Pós-Graduação em Genética e Biologia Molecular, Universidade Federal do Pará, Belém, Brazil; 2 Núcleo de Pesquisas em Oncologia, Universidade Federal do Pará, Belém, Brazil; 3 Laboratório de Análises Clínicas, Instituto de Ciências Biológicas, Universidade Federal do Pará, Belém, Brazil

**Keywords:** Microbiome, Metagenomics

## Abstract

Shifts in subsistence strategy among Native American people of the Amazon may be the cause of typically western diseases previously linked to modifications of gut microbial communities. Here, we used 16S ribosomal RNA sequencing to characterise the gut microbiome of 114 rural individuals, namely Xikrin, Suruí and Tupaiú, and urban individuals from Belém city, in the Brazilian Amazon. Our findings show the degree of potential urbanisation occurring in the gut microbiome of rural Amazonian communities characterised by the gradual loss and substitution of taxa associated with rural lifestyles, such as *Treponema*. Comparisons to worldwide populations indicated that Native American groups are similar to South American agricultural societies and urban groups are comparable to African urban and semi-urban populations. The transitioning profile observed among traditional populations is concerning in light of increasingly urban lifestyles. Lastly, we propose the term “tropical urban” to classify the microbiome of urban populations living in tropical zones.

## Introduction

Gut microbiome metagenomic characterisations across multiple human populations have shed light on the roles of this complex ecosystem in maintaining human health^1,2^. Evidence shows substantial differences in gut/stool microbiome diversity and composition between populations living in diverse subsistence strategies. Generally, individuals living in rural and/or traditional societies harbour highly diverse microbiomes when compared to those from industrialised areas^3,4^. Among other environmental factors, dietary habits, access to medication, sanitation practices and interpersonal contact are mainly responsible for shaping such gut microbial structure^5–7^.

Consumption of highly plant-based diets such as those followed by traditional hunter-gatherers and rural agriculturalists promote gut colonisation by fibre-degrading microbes, such as those from the *Spirochaetes* phylum and *Prevotella* genus^5,8^. For this reason, the gut microbial communities of populations such as the Hazda and Yanomami are regarded as a “window into the past”, given their hosts follow a lifestyle comparable to that of ancient pre-industrialised humans^3,4^. Such a lifestyle is marked by relying on foraging and hunting for food, as well as gender division of labour and seasonal food cycling, markedly opposed to the contemporary industrialised world^9,10^. Thus, it is thought that the gut microbiome of non-urbanised people is ideally adapted to human physiology, as it promotes overall gut health and beneficial interactions with the immune system^11,12^.

Conversely, gut microbial communities of industrialised societies seem to have been altered and are increasingly enriched for mucus-degrading and antibiotic-resistant taxa, which may trigger pro-inflammatory responses and gut dysbiosis^12^. Microbial biomarkers for this lifestyle are typically a high abundance of *Bacteroides* genus and *Akkermansia municiphila*, while diets are rich in animal fat and protein, simple sugars and processed foods^12,13^. Urbanisation and shifts in dietary habits are likely the cause of gut microbial extinctions across generations, disrupting the host–microbiome equilibrium that may eventually lead to the appearance of autoimmune disorders, obesity, type 2 diabetes and other non-communicable diseases^14,15^.

The compositional shifts in gut microbiomes of traditional populations have been a topic of debate and great concern in the field of microbiology and medicine^12^. In the Brazilian Amazon territory, there are 500 Native American populations living across a large urbanisation gradient, with some ethnic groups belonging to a hunter-gatherer and agricultural subsistence lifestyle, while others inhabit areas near small or large urban centres. This subsistence shift will likely result in disrupted gut microbiomes, drawing attention to the dangers of compromising the Amazonian biodiversity present in indigenous settings, which have contributed to health maintenance and ecological balance over thousands of generations^16^.

In this regard, the work of Pires et al.^17^ was the first to characterise the gut microbiome of Brazilian Amazonian populations living in a rural setting. They found that the trade-off between the abundance of *Prevotella* and *Bacteroides* taxa was the main feature distinguishing two Amazonian riverine populations from urban individuals of Rio de Janeiro, located in southeast Brazil. However, these data do not include populations experiencing lifestyle transitions and it remains unclear if these results are transferable to rural Native American Amazonian communities and individuals living in urban Amazonian cities.

Here, we aimed to determine whether the microbiomes of rural Native American populations in the Brazilian Amazon show markers of transition to urbanisation and to what extent recent subsistence changes are impacting gut microbiome compositions. Currently, there are no data comparing the gut microbiome composition of Native American and urban populations from the Brazilian Amazon, a region with vast biodiversity. We employed 16S ribosomal RNA (rRNA) sequencing to profile the gut microbiome of 114 individuals from four distinct populations of urban and Native American Brazilian Amazonians and compared microbial community structures to other urban and rural groups surveyed in Brazil and across the globe.

## Results

### Studied populations and metadata

**Fig. 1:**
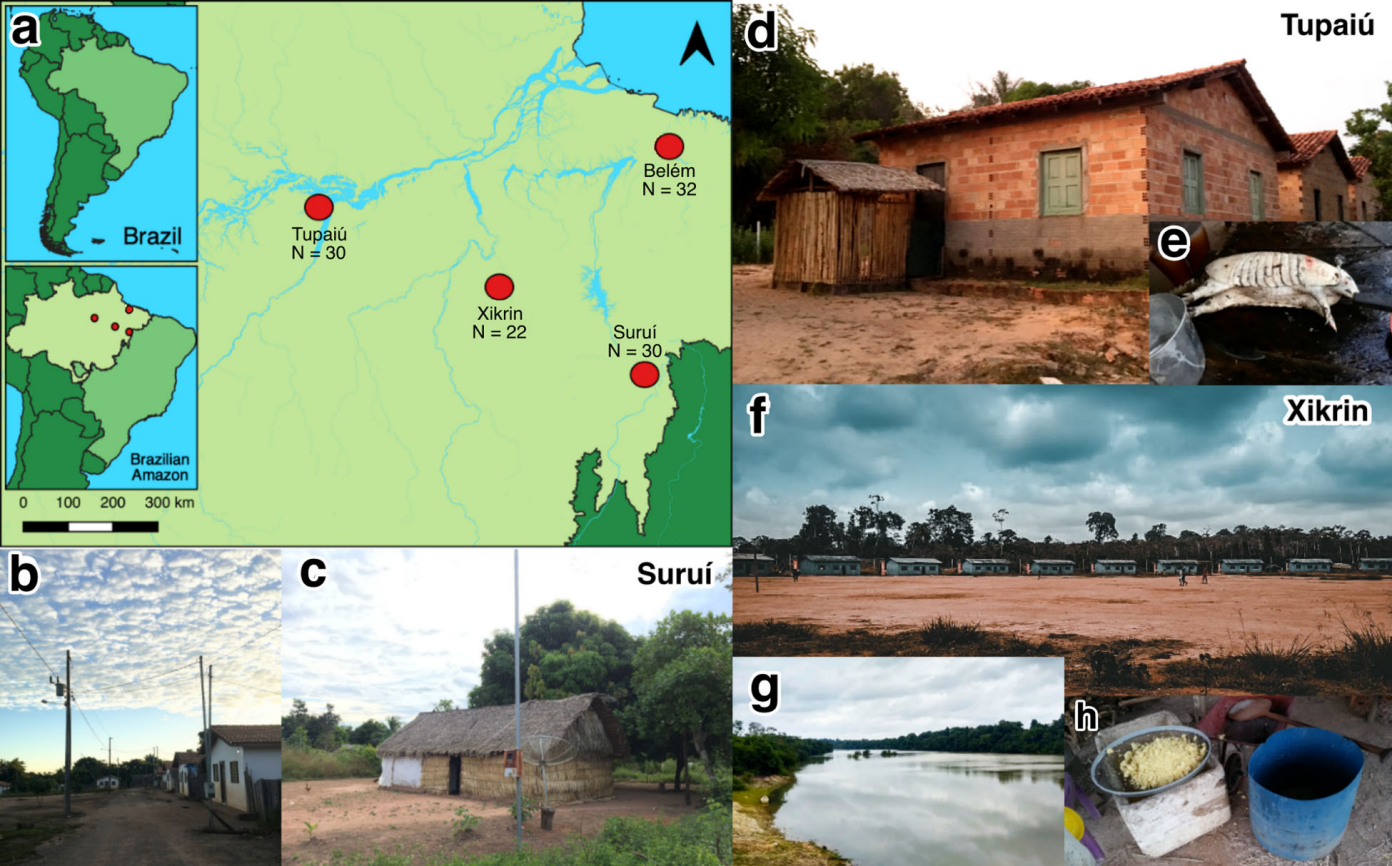
Map and lifestyle patterns of the sampled populations. **a** Location of each sampling site in relation to the South American continent and the Brazilian Amazon. **b**, **c** The village and typical Suruí (R) residence. **d**, **e** Tupaiú (R) home and armadillo being prepared for consumption. **f**–**h** A circular arrangement of Xikrin (R) households, the nearby Bacajá River and the preparation of the cassava flour.

We recruited three rural (R) Native American populations, namely the Xikrin (R) (*N* = 22), Suruí-Aikewara (R) (*N* = 30) and Tupaiú (R) (*N* = 30), and one urban (U) population (Belém, *N* = 32) from the Brazilian Amazon (Fig. 1). A total of 268 and 332 individuals inhabit the indigenous territory of the Xikrin (R) and the Suruí (R) populations, respectively^18,19^. The community located in the extractive reserve home to the Tupaiú (R) houses ~50 families, while over 1.3 million individuals live in Belém (U), the capital of Pará state^20,21^.

Among the Native American communities, access to the Xikrin group is the most logistically challenging, due to the vast and dense Amazon rainforest that composes and surrounds the territory and poor road accessibility. The Suruí are accessible by land, while the Tupaiú are reachable by boat or helicopter, and both are located ~100 km from the nearest rural town. Dietary interviews and metadata collection were used to characterise the subsistence and lifestyle profile of each population (see “Methods” section).

Microscopic examination of faecal samples revealed that 68% of all tested individuals harbour at least one species of gut protozoa, with commensal *Endolimax nana* as the most frequent (Supplementary information 1: Figure S1D). At the population level, the Xikrin (R) had the highest gut protozoa prevalence—except for Belém (U), which only had 35% of samples tested—followed by Suruí (R) and Tupaiú (R) (Pearson’s *χ*^2^ test, *p* value = 0.029) (Supplementary information 1: Figure S1F). Gut helminths were observed in 6% of samples, belonging to either Xikrin (R), Tupaiú (R) or Belém (U) populations. Regarding *Entamoeba* sp. colonisation, 20% of samples tested positive for *E. coli*, *Entamoeba histolytica/dispar* or both. Of these, 39% were Xikrin (R), 30% were Tupaiú (R), 18% were Suruí (R) and 13% from Belém (U).

### Amazonian rural and urban microbiomes show similar diversities

**Fig. 2:**
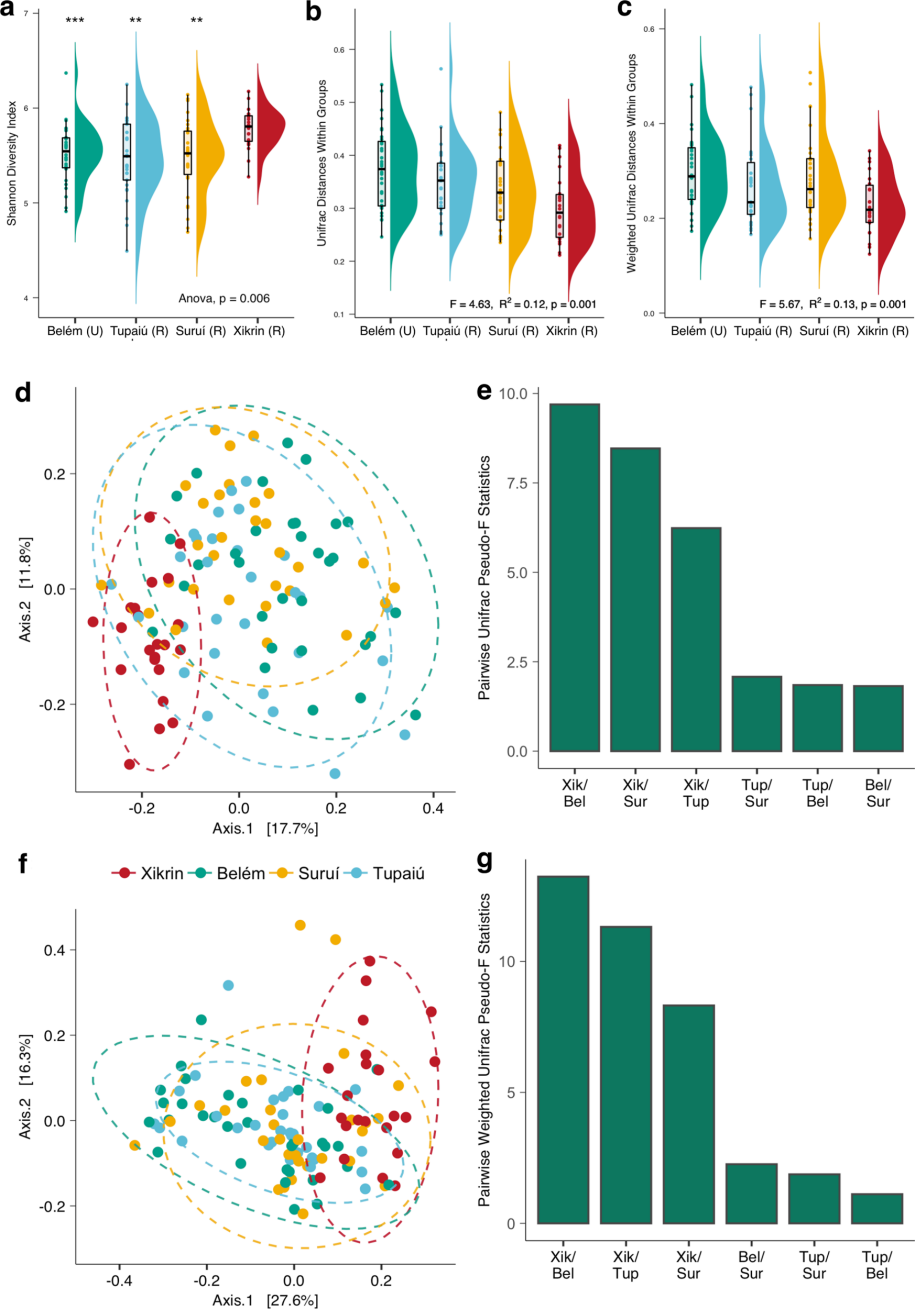
Within- and between-group diversity measures. **a** Shannon index. **b** Unweighted Unifrac distances within groups. **c** Weighted Unifrac distances within groups. **d** PCoA of unweighted Unifrac distances. **e** Weighted Unifrac pairwise PERMANOVA differences among population pairs. **f** PCoA of weighted Unifrac distances. **g** Unweighted Unifrac pairwise PERMANOVA differences among population pairs. *NS* not significant, *p ≤ 0.05, **p ≤﻿ 0.01, ***p ≤﻿ 0.001, ****p ≤ 0.0001.

Alpha diversity was computed to determine differences in richness estimates among the Amazonian populations (Supplementary Data 1: Table 1). This analysis revealed that the Xikrin (R) harbour the most diverse and Tupaiú (R) the least diverse microbiomes among the investigated groups (Shannon index analysis of variance (ANOVA), *p* value = 0.006) (Supplementary information 1: Figure S2). The urban population from Belém had the second highest Shannon diversity index mean and similar diversity to Suruí (R) and Tupaiú (R) traditional populations (Fig. 2a).

Spearman’s correlation analyses (Supplementary information 1: Figure S3A–D) did not show significant associations among alpha-diversity values and age or sex data for any of the populations (*R* = −0.023, *p* values = 0.71, Shannon index for “age” and *T* test, *p* value = 0.13 for “sex”). However, higher alpha-diversity values were associated with the presence of gut protozoa colonisation as showed by Shannon and Chao1 diversity indexes (*T* test, *p* value = 0.036 and 0.008, respectively) and number of observed species (*T* test, *p* value = 0.0099) (Supplementary information 1: Figure S3A–D). Further, concurrent colonisation by *Escherichia coli* and *Entamoeba histolytica*/*dispar* was associated with higher Chao1 alpha diversity (ANOVA, *p* value = 0.041) and number of observed species (ANOVA, *p* value = 0.042) (Supplementary information 1: Figure S4R–T).

Further, although no statistical significance was detected (*T* test, *p* value = 0.051), we also observed that individuals with helminth intestinal colonisation had increased alpha-diversity values when compared to negative microscopic examinations (Supplementary information 1: Figure S4M–P).

To assess similarities among microbial community structures, we computed beta diversity with Unifrac and Weighted Unifrac distances. Results show that the Xikrin (R) population shares the most compositional features, followed by Suruí (R), Tupaiú (R) and Belém (U). We also used permutational multivariate analysis of variance (PERMANOVA) to test whether the dispersion from centroid values was the same among all groups. In this analysis, rural microbiomes showed less inter-individual variation when compared to the urban group (PERMANOVA *p* value = 0.001) (Fig. 2b, c). No significant correlation between alpha- and beta-diversity measures were observed (*R* = −0.74, *p* value = 0.26) (Supplementary information 1: Figure S5A–H).

Principal coordinate analysis (PCoA) based on Unifrac and Weighted Unifrac distances revealed that the Xikrin (R) population, although with a small degree of overlap, forms a distinct cluster and shows far less dispersal than other population groups, which indicates a more homogeneous microbiome structure among individuals (Fig. 2d, f). Further, this analysis highlights the structural similarities between rural and urban groups, demonstrated by the considerable overlap between the Suruí (R), Tupaiú (R) and Belém (U) samples. In the computed pairwise PERMANOVA analysis on Unifrac and Weighted Unifrac distances (Fig. 2e, g), the greatest amount of variation was among Xikrin (R) and Belém (U), followed by Xikrin (R) and all other population groups. Surprisingly, the least amount of variation was observed between pairs Belém (U) and Suruí (R), and Belém (U) and Tupaiú (R) populations, suggesting there are little compositional differences between such microbial communities.

### Microbial compositions are shared among rural and urban groups

Considering we did not find discrete population clustering between all rural and non-rural dwellers, we sought to identify which taxonomic features could be driving compositional similarities observed in Unifrac distance-based PCoA analyses. First, we determined the most frequent genus in each individual microbiome, characterised by the taxa with the highest relative abundance in each sample (Supplementary information 1: Figure S6A). Nine taxa summarise the most frequent genera across all samples: *Prevotella* (53%), *Faecalibacterium* (14%), *Bacteroides* (9%), *Roseburia* (9%), *Succinivibrio* (7%), *Treponema* (5%), *Oscillospira* (2%), *Escherichia* (1%) and *Ruminobacter* (1%).

Notably, aside from microbiomes with *Escherichia*, *Bacteroides* and *Faecalibacterium* as the most frequent genera, nearly all other samples had *Prevotella* genus in the top three most prevalent taxa. For individuals with *Faecalibacterium*-prevalent microbiomes, more than half harboured *Prevotella* as one of the top three most frequent taxa.

*Prevotella* is the most frequent dominant taxa in all populations (Supplementary information 1: Figure S6A). Nonetheless, we observed typically urban-like signatures in both urban and rural samples, aside from Xikrin (R). For instance, *Bacteroides*- and *Faecalibacterium*-prevalent microbiomes were observed only in Belém (U), Suruí (R) and Tupaiú (R), while the presence of individuals with *Treponema* as the most prevalent taxa were only observed among the Xikrin (R) and Suruí (R).

Further, considering higher abundance of *Bacteroidales* in relation to *Clostridiales* order is a biomarker for traditional microbiomes from Africa and South America^4,22^, we tested such proportions across sampled Amazonian populations. The Xikrin (R) presented a higher relative proportion of *Bacteroidales* when compared to other populations (ANOVA test, *p* value = 4.7e − 08), while other Native American groups are not significantly different from urban Belém (U) (Supplementary information 1: Figure S6B).

**Fig. 3:**
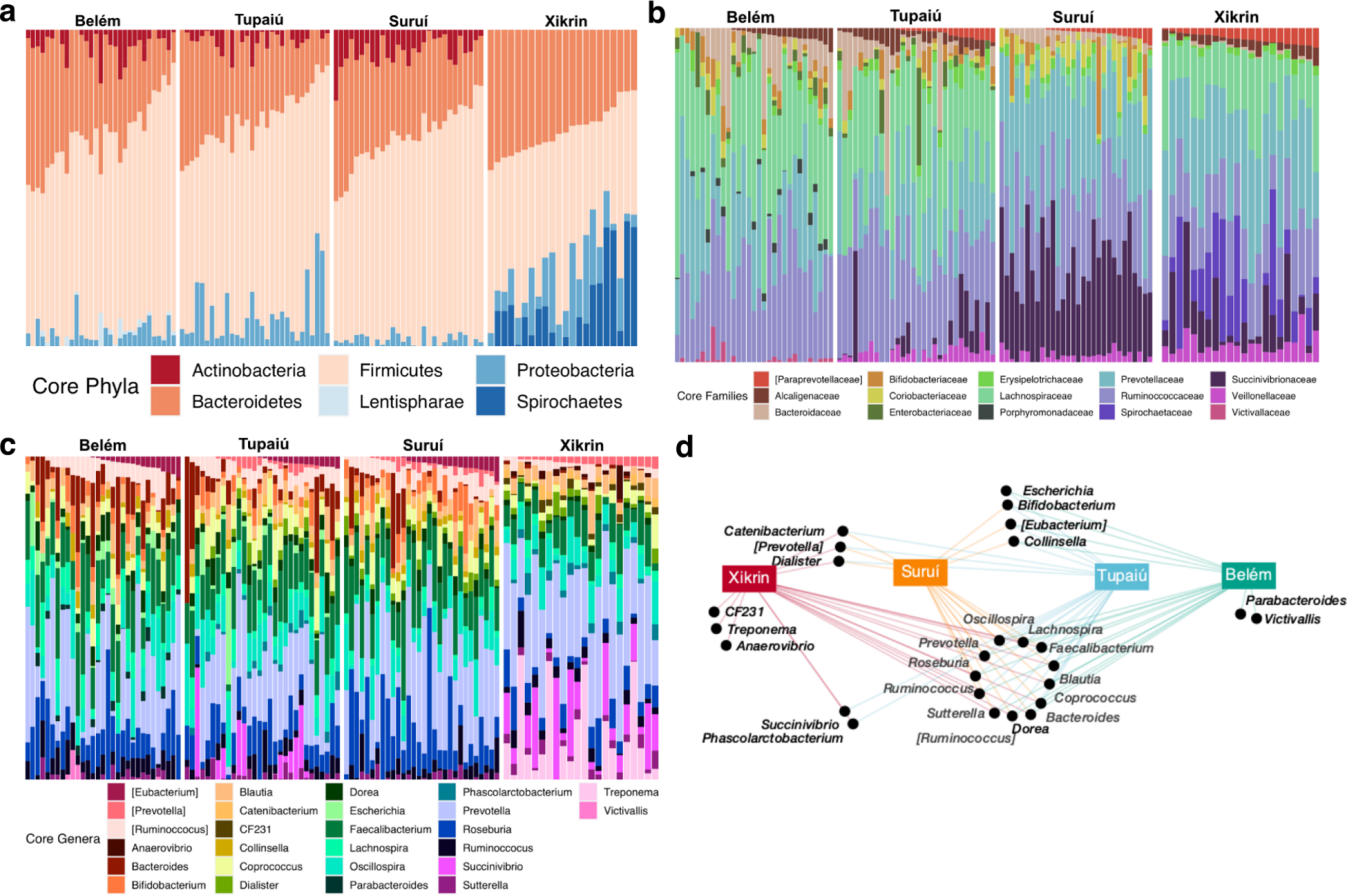
Core microbiome distribution. **a** Abundance of core microbiome phyla per population. **b** Abundance of core microbiome families per population. **c** Abundance of core microbiome genera per population. **d** Network of the shared core microbiome genera in each population.

Moreover, an analysis of the population-based core microbiome was performed to explore taxonomic compositions shared between individuals from the same community. At higher taxonomic resolutions, such as phyla, core taxonomic compositions and proportions are similar among Belém (U), Suruí (R) and Tupaiú (R), with abundance variability displayed mainly within Proteobacteria taxa and the presence of Lentispharae in Belém (U) (Fig. 3a). The Xikrin (R) population was distinct for the presence of Spirochaetes and the absence of Actinobacteria in the core microbiome.

Lower taxonomic levels such as family and genus barplots (Fig. 3b, c) showed substantial similarities between Belém (U) and Tupaiú (R), in which the proportions of *Ruminococcaceae*, *Lachnospiraceae* and *Prevotellaceae* are highly comparable. Nonetheless, almost all Tupaiú (R) participants show the presence of *Veillonellaceae* and approximately half harbour variable abundances of *Succinivibrionaceae*, a feature shared only by other sampled Native Americans. For instance, the highest abundances of *Succinivibrionaceae* belong to Suruí (R), while the presence of *Spirochaetaceae* is unique to the Xikrin (R), represented by *Treponema* at the genus level.

Further, network analyses showed that 46% of core genera are shared among all populations and 80% are shared by at least two groups (Fig. 3d). Interestingly, only Belém (U) and Xikrin (R) showed group-specific genera: *Parabacteroides* (Bacteroidetes) and *Victivallis* (Lentisphaerae) in Belém (U), and *CF231* (Bacteroidetes), *Treponema* (Spirochaetes) and *Anaerovibrio* (Firmicutes) in Xikrin (R). Hypergeometric enrichment *p* values were analysed and indicated significant overlaps between the core microbiomes of the Suruí (R) and Tupaiú (R) populations (BH *p*-adj value = 2.2e − 3) (Supplementary Data 1: Table 2).

Next, we investigated microbial co-occurrence patterns to determine relationships between different genera in the microbiome ecosystem. Using sparse correlation coefficients (SparCC)^23^, we found two main co-abundance groups that seem to be centred around the trade-off between *Prevotella* and *Bacteroides* (Supplementary information 1: Figure S7A–C). In the *Prevotella* co-abundance group, other taxa such as *CF231*, *Treponema*, *Succinivibrio* and *Catenibacterium* are seen in high frequencies. In contrast, mainly *Odoribacter* and *Escherichia* genera were observed to have comparable frequencies to *Bacteroides* in the opposite co-abundance group. The optimal number of clusters in this analysis was assessed by *k*-means and Partitioning Around Medoids (PAM) clustering methods and validated with Hopkins statistic (Supplementary information 1: Figure S7B).

### Signs of transition to urbanisation among Amazonian microbiomes

Given that Native American populations (Suruí and, especially, Tupaiú) share more similarities with Belém (U) than the rural, more remote Xikrin, three scenarios were suggested: (1) Suruí (R) and Tupaiú (R) populations are increasingly displaying urban-like microbiomes, hence their shared features with Belém (U); (2) Belém individuals do not follow a typically urbanised microbiome composition and are more similar to other non-urban human groups, or (3) both scenarios are occurring simultaneously.

**Fig. 4:**
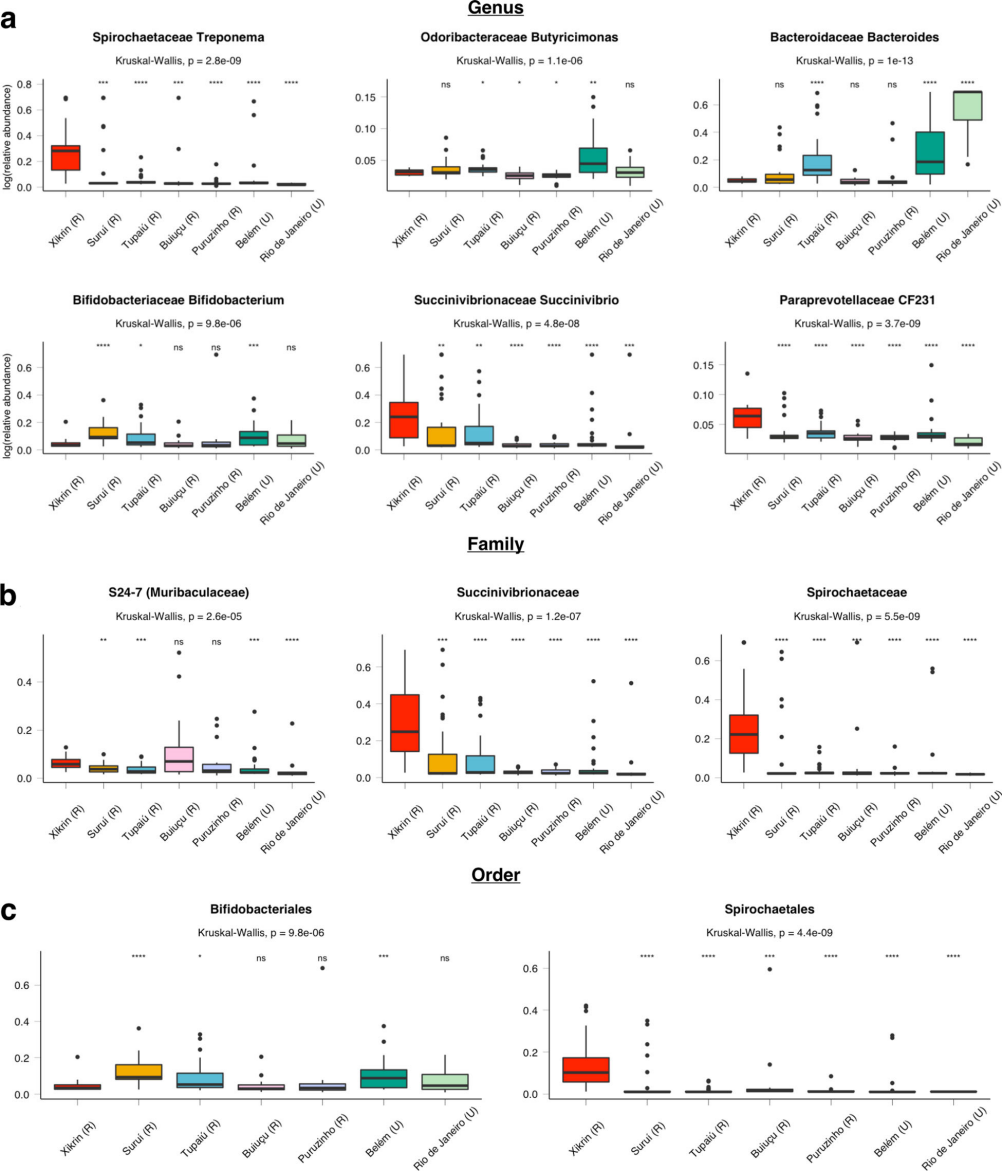
Differentially abundant taxa among Brazilian populations as determined by ANCOM analyses considering a *W* > 0.9 cut-off significance value. **a** Differentially abundant genera. **b** Differentially abundant families. **c** Differentially abundant orders. *NS* not significant, *p ≤ 0.05, **p ≤ 0.01, ***p ≤ 0.001, ****p ≤ 0.0001.

To test these hypotheses, we computed significant differentially abundant taxa at genus, family and order taxonomic resolutions with analysis of composition of microbiomes (ANCOM)^24^ between populations from the present cohort and compared results to others from Brazil: the rural Amazonian riverine Buiuçu (R) and Puruzinho (R) populations and urban individuals of Rio de Janeiro (U)^17^ (Fig. 4). Results are shown as the logarithm of re-scaled relative abundances, as described by Hansen et al.^22^.

ANCOM results showed that six taxa are differentially abundant between groups at the genus level (Fig. 4a). Of these, *Treponema* (Kruskal–Wallis, *p* = 2.8e − 09), *Succinivibrio* (*p* value = 4.8e − 06), and *CF231* (*p* value = 3.7e − 09) were more abundant in the Xikrin (R) and display decreasing abundances according to urbanisation. In contrast, *Butyricimonas* showed the highest abundance in Belém (U) (*p* value = 1.1e − 06), while *Bacteroides* was most abundant in Rio de Janeiro (U), Belém (U) and Tupaiú (R) (*p* value = 1e − 13). *Bifidobacterium* (*p* value = 9.8e − 06) showed the lowest abundances among three rural communities (Xikrin, Buiuçu and Puruzinho) and the urban Rio de Janeiro.

At the family level, differential abundance analyses detected *S24-7* (*Muribaculaceae*) as significantly more abundant in the microbiomes of the Xikrin (R), Buiuçu (R) and Puruzinho (R) (Kruskall–Wallis, *p* value = 2.6e − 05). Moreover, *Succinivibrionaceae* and *Spirochaetaceae* families were significantly more abundant among the Xikrin (R) when compared to other tested populations (Fig. 4b). Lastly, the *Bifidobacteriales* (Kruskal–Wallis, *p* value = 9.8e − 06) and *Spirochaetales* (Kruskal–Wallis, *p* value = 4.4e − 09) orders were found to be differentially abundant. Interestingly, the abundance of *Bifidobacteriales* among the Xikrin, Buiuçu and Puruzinho rural groups was not significantly different from urban Rio de Janeiro, but was significantly lower than remaining rural populations (Fig. 4c).

We also computed differential abundance analyses with South American native and rural populations (Supplementary information 1: Figure S8)^4,5^. ANCOM boxplots show that *Prevotella* abundances in Brazilian Amazonian individuals, particularly the Xikrin (R), are similar to those found for Venezuelan Yanomami (R) and the Tunapuco from the Peruvian Andes (R) populations.

Differential abundances for the Amazonian populations in this cohort compared to African populations living in a gradient of urbanisation in Cameroon^7^ (Supplementary information 1: Figure S9) revealed that *Prevotella* abundances in Amazonian populations are similar to what is observed in an urbanisation transitioning context. For instance, Xikrin (R) abundances of this genus are similar to that of rural Ngoantet, while Suruí (R) and Tupaiú (R) show abundance means comparable to semi-urban Mbalmayo. The lowest *Prevotella* abundance in this comparison belongs to the urban Yaounde, the capital of Cameroon, while Belém (U) has the lowest among Amazonian groups, yet higher than the Yaounde population.

This pattern is the same for the *Bacteroides* genus, but with inversed proportions. When observing the *Succinivibrio* genus, this tendency continues with the exception of the Suruí (R) and Tupaiú (R) populations, which display mean abundances more comparable to urban than the semi-urban and rural groups. However, some genera (*e.g. Oscillospira*, *Coprococcus* and *Mitsuokella*) have higher abundances in Brazil than in Cameroon, independently of urbanisation levels.

**Fig. 5:**
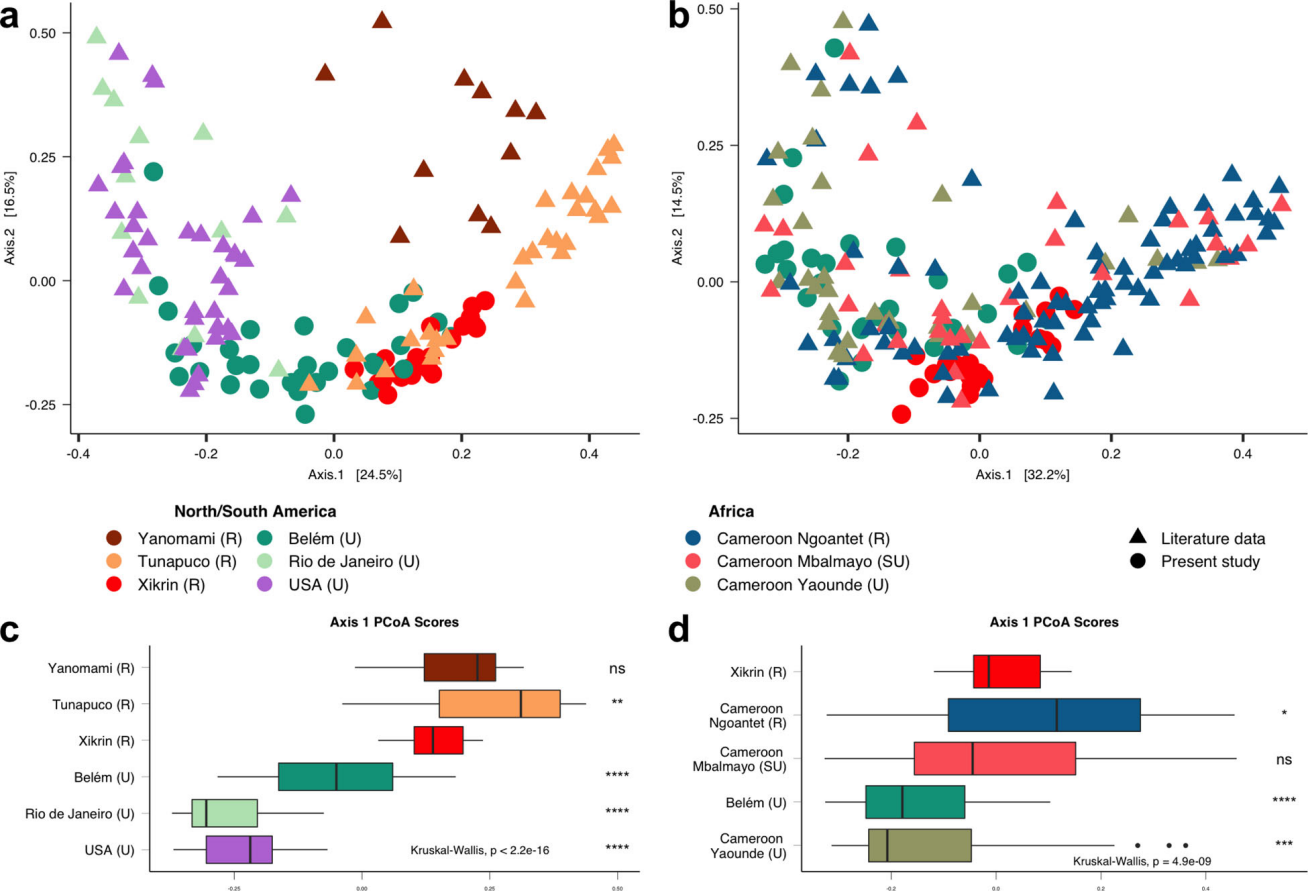
PCoA of Bray–Curtis distances. **a** Distances between Amazonian Xikrin (R) and Belém (U) populations to other groups from the American continent. **b** Distances between Amazonian Xikrin (R) and Belém (U) populations to African groups from an urbanisation gradient in Cameroon. **c** Boxplots of PCoA scores along axis 1 for the American continent beta-diversity analysis. **d** Boxplots of PCoA scores along axis 1 for the African urbanisation gradient beta-diversity analysis. *NS* not significant, *p ≤ 0.05, **p ≤ 0.01, ***p ≤ 0.001, ****p ≤ 0.0001.

Using PCoA based on Bray–Curtis distances to compare the Xikrin (R) (most rural group) and Belém (U) (most urbanised) populations with other South American and USA populations, we found that the Brazilian Amazonians were located at the intermediate stage of an urbanisation gradient (Fig. 5a, c). The Peruvian Tunapuco (R) largely overlap the Xikrin (R) and some Belém (U) individuals, while others from Belém (U) clearly share features with urban populations.

The same analysis was used to compare the Xikrin (R) and Belém (U) populations to three Cameroonian populations living in a gradient of urbanisation. We found that Belém (U) largely overlaps with Yaounde (U) and Mbalmayo (SU) populations, while the Xikrin (R) are similar to both the Ngoantet (R) and the Mbalmayo (SU) (Fig. 5b, d). Additional comparisons to African rural populations, such as the Tanzanian Sandawe and the Botswana San, can be found in Supplementary information 1: Figure S10.

In light of the various technical differences across gut microbiome datasets, meta-population comparisons must be interpreted with caution as they do not rule out the influence of technical factors in producing such findings.

### Different predicted metabolic functions among urban and traditional groups

We used Phylogenetic Investigation of Communities by Reconstruction of Unobserved States (PICRUSt) to predict the metabolic potential of microbial communities based on 16S data and Kyoto Encyclopaedia of Genes and Genomes (KEGG) pathways^25^.

**Fig. 6:**
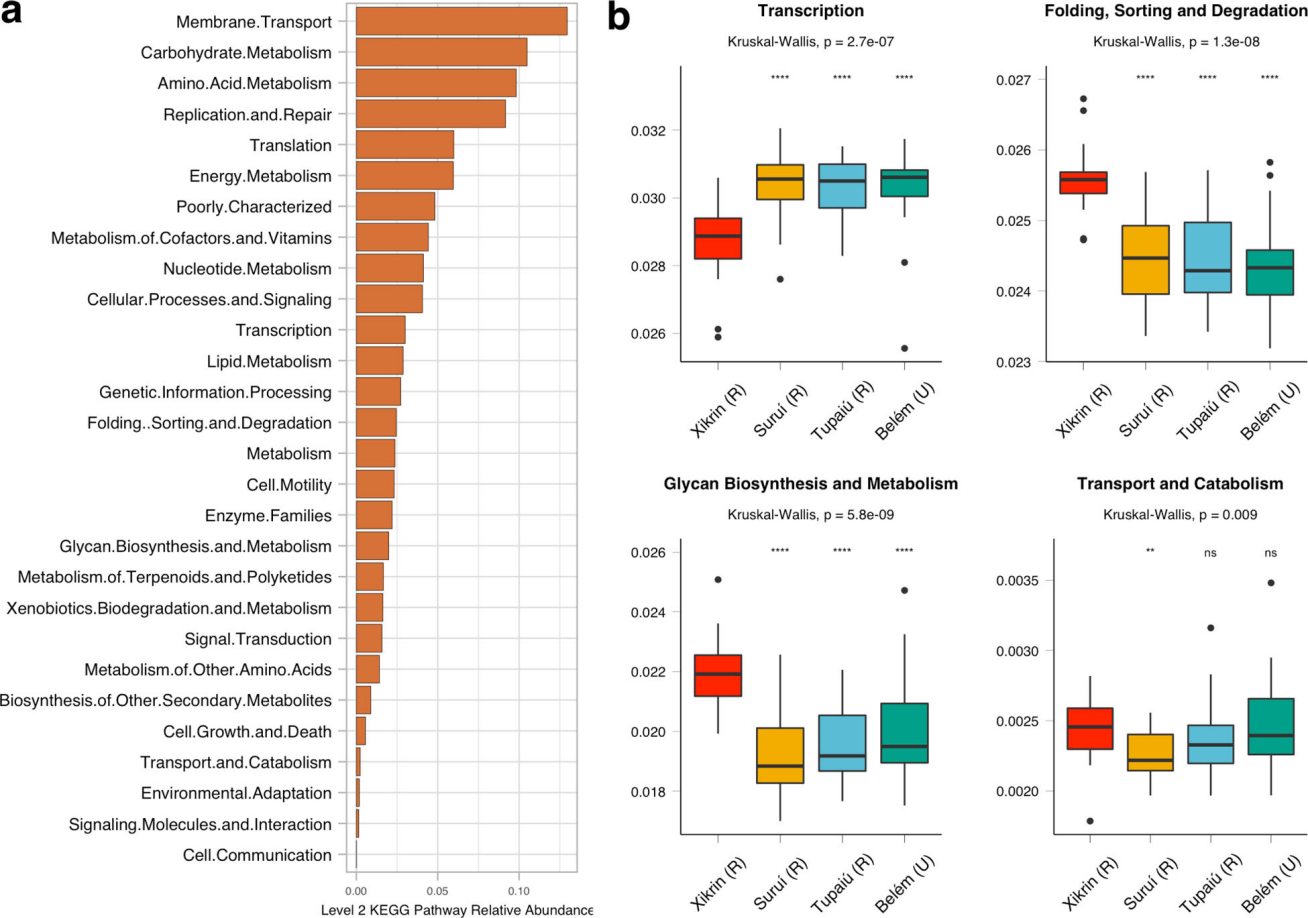
Functional predictions based on PICRUSt results. **a** Relative abundance of level 2 KEGG pathways including all samples. **b** Differentially abundant KEGG pathways as determined by ANCOM (*W* > 0.9). *NS* not significant, *p ≤ 0.05, **p ≤ 0.01, ***p ≤ 0.001, ****p ≤ 0.0001.

After removing eukaryote-related pathways, PICRUSt results identified 30 different metabolic functions summarised to level 2 KEGG pathway resolutions (Supplementary Data 1: Table 6). Pathways related to membrane transport, carbohydrate metabolism, amino acid metabolism, replication and repair, translation and energy metabolism were responsible for 54% of the observed metabolic predictions (Fig. 6a).

Then, we performed ANCOM to identify which pathways were differentially abundant among the Amazonian populations (Fig. 6b). Considering a *W* > 0.9 significance threshold, ANCOM results revealed four pathways, namely transcription, glycan biosynthesis and metabolism, folding, sorting and degradation, and transport and catabolism. Of these, glycan metabolism and folding, sorting and degradation were enriched among the Xikrin (R) population, while transcription was found to be significantly decreased. Finally, the transport and catabolism pathways were significantly less abundant among the Suruí (R).

When considering significance levels at *W* > 0.6, ANCOM revealed 27 differentially abundant pathways (Supplementary Data 1: Table 7). Here, we highlight that pathways related to carbohydrate metabolism (Kruskal–Wallis, *p* value = 1.4e − 07) and xenobiotics biodegradation (Kruskal–Wallis, *p* value = 0.0012) were found significantly more abundant among in Belém (U) microbiomes, and decreased according to the urbanisation gradient (Supplementary information 1: Figure S 11G, O).

## Discussion

We have characterised the gut microbiome of several populations living in diverse lifestyles in the Brazilian Amazon. Our findings support a pattern of transition to urbanisation among Native American populations living outside urban centres, showing that gut microbiomes of rural Native American people are experiencing structural modifications and these data represent a glimpse into the stages of biodiversity loss and/or microbial compositional transitions during such changes.

Higher alpha-diversity values for the Xikrin (R) were expected, as traditional populations far from industrialisation have been shown to display higher microbial diversity than industrialised individuals^1,3,4,22^. Further, the Native American Suruí (R) and Tupaiú (R) displayed similar diversity to that of urban Belém, which is comparable to those previously observed among Rio de Janeiro and Amazonian riverine individuals^17^.

A compositional transition among the rural populations of Suruí and Tupaiú is further supported by the high rate of dispersal in beta-diversity distance measures, similar to that of an urban group such as Belém. Urbanisation evidence is also demonstrated by the distribution of the most prevalent genera in individuals of each group (Supplementary information 1: Figure S6A). For instance, *Treponema*-prevalent microbiomes only in the Xikrin and the Suruí may reflect that the Suruí are more recently changing lifestyle patterns, as these taxa are considered a biomarker of traditional societies^3–5,12^.

The Xikrin (R) follow a diet mostly composed of highly fibrous tubers such as sweet potatoes and cassava, which explains the abundance of polysaccharide degrading taxa in the gut microbiome, as reported for other traditional groups such as the Yanomami, Matses, Hazda and BaAka^3–5,8^. Meanwhile, the absence of *Treponema* prevalence among the Tupaiú (R) could indicate that a transition to an urbanised microbiome is more advanced, reinforced by the higher frequency of *Bacteroides*-prevalent microbiomes. Further, the abundance of *Bacteroides* and *Bifidobacterium* taxa have shown to be antagonistic to the colonisation of *Treponema* species in urbanised individuals, representing an adaptive response to increased consumption of refined carbohydrates and dairy^26^.

The fact that only Xikrin (R) and Belém (U) core microbiomes showed group-specific taxa (genera not present in core microbiomes of other populations), such as *Treponema* and *Parabacteroides*, respectively, suggests that they represent opposite extremes of the urbanisation gradient in the Amazonian gut microbiomes (Fig. 3D). However, it is noteworthy that the urban Amazonian microbiome is different and should not be regarded as a typically urban-like profile such as what is shown for the United States, Italian and other industrialised societies^5,17,27^. This is especially evidenced by the similarities shared by Belém (U) and urban and semi-urban groups from Cameroon^22^ and its differences from USA and Rio de Janeiro individuals (Fig. 5a).

Despite being the capital of the Pará state and populated by nearly two million people, Belém is an urban centre in which only 14% of the population receives sewage collection and, of these, only 3% are treated^28^. Moreover, the city is located at the margins of the Pará river and in close proximity to the rainforest environment, which may have an important role in microbial dispersal and composition. Such factors may explain the prevalence of gut protozoa and alpha-diversity values among the urban individuals in our study. In addition, the dietary habits of Belém individuals are unique, as they consist of industrialised food consumption while also including fresh and unprocessed items such as cassava flour, açaí and tropical fruits regularly. This diet may have an effect on the fibre intake of this population, explaining the abundance of *Prevotella* among such individuals.

We argue that diet, geographical proximity to native biodiversity and lack of adequate access to sanitation has an important role in determining the gut microbial composition of Belém individuals, in spite of subsistence strategy. Thus, we propose the term “Tropical Urban” as a category of subsistence strategy, in the scope of microbiome research, to define populations inhabiting urban settings located in the tropical zone. This new classification considers that tropical urban environments are inserted in a context of proximity to great biodiversity, which has an important impact in defining the ecological and cultural contexts of these cities. Such circumstances determine interactions with microbial diversity and, consequently, shape gut microbial compositions, as seen for Belém and its similarities to urban African populations.

Further, we clarify that the term “semi-urban” is not an appropriate categorisation of these areas, as they constitute the urban extreme in the local gradient of urbanisation. Additional comparisons to African and Asian populations living in tropical urban settings will elucidate which aspects of such environments most influence gut microbial compositions.

Our data corroborate recent findings of gut eukaryotes being one of the main factors involved in microbiome alpha diversity among neighbouring rural and urban populations^7^. In the case of the present Amazonian populations, however, alpha diversity was influenced only by concurrent infections of *Entamoeba coli* and *E. histolytica/dispar*. As discussed by Lokmer et al.^7^, an industrialisation transition is accompanied by a loss in bacterial diversity and colonisation by *Entamoeba* sp. If we apply this concept to our data analysing overall *Entamoeba* sp. prevalence, an industrialisation gradient would have the Xikrin (R) at one side of the spectrum with increasing industrialisation passing by Suruí (R) Tupaiú (R) and Belém (U) (Supplementary information 1: Figure S1E).

Since we did not obtain individual dietary information for the Native American populations, we were not able to make direct correlations between microbial composition and dietary habits. However, considering we found varying degrees of urbanisation in the microbiomes of rural populations, it is possible that factors, such as a non-sexual subdivision of labour, play a part in determining gut microbial composition, simitar to what has been described for the Hazda hunter-gatherers^3^.

We hypothesise that individuals responsible for periodically leaving the indigenous territory for commercial or social affairs in close-by semi-urban towns may display more urbanised gut microbiomes when compared to those in charge of tending to crops, for instance. This access to a semi-urban environment may increase the consumption of industrialised foods and exposure to antimicrobial medications, driving compositional changes in gut microbial communities. Future studies controlling for such community roles as well as longitudinal studies will be helpful in determining the conditions under which the gut microbiomes of rural Native American people are more prone to urbanisation.

The transition gradient observed in the present cohort may, therefore, be driven by factors that influence access to an urbanised environment in general. For instance, access to the Xikrin (R) community is the most difficult, which makes it troublesome (but not impossible) to obtain urban food products. This is not the case for the Suruí (R) and Tupaiú (R), although the latter is only accessible by boat or helicopter. Moreover, language is expected to impose a barrier for transitioning. The Xikrin (R) are part of the Jean linguistic group, and only a select number of individuals speak Portuguese. Conversely, the Suruí (R), although having non-Portuguese speakers, has a larger number of individuals who were able to communicate with the research staff. Regarding Tupaiú (R), their native language was hardly spoken, considering they are a mixture of multiple Native American ethnicities.

Language may also stand as a genetic obstacle, meaning non-Portuguese speaking communities are possibly more ethnically homogeneous. It is possible, therefore, that a language barrier is one of the causes for little to no evidence of urbanisation among the Xikrin gut microbiome, as substantial and continued contact with urbanised populations is rare for a majority of these individuals. Therefore, we suggest that future research should investigate host genetic diversity as a means of elucidating its role in the urbanisation transition on gut microbiomes.

In this sense, we highlight that ethnicity has also been found to play a role in determining gut microbiome structures, as it can serve as a proxy for dietary and lifestyle variability^29^. Considering the urban population of Belém is ethnically diverse^30^ and the Tupaiú are made up of individuals from multiple Native American ethnicities^31^, it is possible that this may influence the high inter-individual variability observed among the Belém and Tupaiú populations. This is further supported by the homogeneous ethnicity of the Xikrin, which also displayed homogeneous gut microbial profiles, but is contradicted by the microbial variability seen among the Suruí, which are not ethnically diverse^32^.

Regarding clustering analyses with other South American traditional populations, the Xikrin (R) showed more proximity to the Andean Tunapuco^5^ than to the recently contacted Yanomami of Venezuela^4^. Despite both Yanomami and Xikrin being Amazonian native communities, the Yanomami follow a hunter-gatherer lifestyle, while the Xikrin are adapted to an agricultural system. In addition, this might explain the separation between the Xikrin and other hunter-gatherers such as the Bostwana San and the Tanzania Sandawe^22^ (Supplementary information 1: Figure S10A), and their similarities with the Tunapuco population, which also presents rural agriculture subsistence strategies (Fig. 5a)^5^. It is important, however, to state the possible bias in interpreting meta-population microbiome data given the technical differences employed in the generation of each dataset.

Nonetheless, compared to other rural populations, all the Amazonian samples analysed in this study showed higher abundances of *Coprococcus*, *Lachnospira* and *Sutterella* taxa (Supplementary information 1: Figure S8). A similar abundance of such groups was found to be associated with urban populations of Burkina Faso and Italy^27^. *Lachnospiraceae* family seems to be associated with non-communicable diseases such as obesity and metabolic syndrome^33^, while its *Coprococcus* genus has also been associated with low high-density lipoprotein concentrations, high blood pressure and hyperglyceridaemia across the epidemiological transition^34^. Members of the *Lachnospiraceae* family (such as *Coprococcus*) were also found to be enriched among the Native American Cheyenne and Arapaho individuals from North America, which are increasingly shifting towards an industrialised lifestyle pattern^35^. Considering a recent report^36^ showing increased prevalence of obesity and type 2 diabetes among the Xikrin (R), the abundance of these taxa points to a gut microbiome similar to that of urbanised populations even in an environment with minimal intake of processed foods and medication, difficult access and linguistic barriers. In an Amazonian rural Native American population, this double burden of diseases can lead to public health issues.

An urbanisation of the gut microbiome of rural populations is also corroborated by PICRUSt metabolic pathway predictions. Despite limitations of this approach, predictions show an increasing abundance of pathways linked to urbanised microbiomes among Amazonian populations, such as membrane transport, carbohydrate metabolism and xenobiotics biodegradation, similar to what has been seen for Bantu, Tanzania and Botswana populations undergoing urbanisation^8,22^ As discussed by Gomez et al.^8^ for the Bantu individuals, such pathways are associated with higher exposure to pesticides as well as food additives, frequently present in industrialised and processed foods.

The gut microbiome characterisation of heterogeneous traditional/rural and urban communities from the Amazon represents the opportunity to observe the worldwide tendency of changes in gut microbiome composition and transitions in an environment known for its tremendous biodiversity. We observe a local and global transitioning gradient among such populations, with the Native American Xikrin (R) as the most rural-like microbiome and similar to that of other agricultural South American societies. Suruí (R) and Tupaiú (R) show a transitioning microbiome with both signs of traditional and industrialised communities, and the urban Belém (U) was similar to the urban and semi-urban African population.

It is critical that we promote the inclusivity of diverse populations in microbiome research to allow that all human groups benefit from scientific/clinical advancements. The increasing rhythm observed in the prevalence of metabolic diseases and its association with gastrointestinal microbiomes makes the characterisation of Amazonian gut microbiomes an urgent topic in terms of public health. Further studies should investigate a longitudinal perspective for tracking the transition process while controlling for disease biomarkers and host genetic factors.

## Methods

### Ethics approval

Ethics approval and indigenous territory entry permits were obtained through the Research Ethics Council from the Federal University of Pará and from the National Council on Research Ethics, under protocol number 3.094.486. Written informed consent was obtained from the urban-living participants individually and a group consent was obtained from the ethnic leadership in each Native American community, as established in the Brazilian legislation for research in Native American communities (CNS 304/2000). This research was carried out according to the ethical principles established by the Declaration of Helsinki .

### Population characterisation

We sampled 114 individuals from four locations in the Brazilian Amazon including rural Native American communities (Xikrin, *N* = 22, Suruí, *N* = 30 and Tupaiú, *N* = 30) and one urban population (Belém, *N* = 32). All three Native American communities were visited during the dry season in the Amazon rainforest, which spans from May to December. Figure 1 shows population locations in relation to South America and the Brazilian Amazon. Maps were designed by the authors in QGIS v.3.18 using geographical limits from IBGE public database^37,38^.

Access to the Trincheira-Bacajá indigenous territory, home to the Cateté Xikrin and Bacajá Xikrin ethnic groups, is the most difficult as it requires over 12 h of land and river travel through the dense Amazonian rainforest, characterising it as the most remote of the sampled populations. The Cateté and Bacajá Xikrin people inhabit opposing sides of the territory and are named according to nearby rivers. In this study, we included the Bacajá Xikrin individuals (here referred to as Xikrin), who inhabit the margins of the Bacajá River. Subsistence practices among the Xikrin include mostly subsistence agriculture (sweet potatoes, cassava, corn, pumpkin and bananas), small game hunting, fishing and gathering of nuts and fruits. The Xikrin are known for hunting and gathering over long distances across the territory, which allows for a great food variety.

The Suruí live in the Sororó indigenous territory and access is done by land; the villages are located ~100 km from the closest rural town. Subsistence modes among the Suruí consist of small-scale cattle raising, rice cultivation and small game hunting, and is progressively less dependent on subsistence agriculture, although it is still present mainly through cassava root and sweet potatoes. Industrialised food products such as frozen poultry, sugar, dairy and crackers are increasingly common.

The Tupaiú are one of the several emergent Native American people that inhabit the Tapajós-Arapiuns Extractive Reserve, located by the margins of the Tapajós River, a major tributary of the Amazon River^31^. Access to this territory is only possible by boat or helicopter. Nevertheless, communication among neighbouring villages is common, and communities are frequently formed by people from multiple ethnic backgrounds. Located in a riverine setting, the subsistence practices are largely dependent on fishing, small game, cassava root agriculture and fruit harvesting.

The urban population from Belém was recruited in the Federal University of Pará (UFPA) and samples consisted of university students, faculty members and surrounding neighbourhoods. Belém is the capital and largest city of the Pará state, in the northern region of Brazil, and is located at the mouth of the Amazon River. The typical diet reported by participants consists mainly of rice, beans, animal protein, manioc flour, dairy products and industrialised foods.

### Sampling and metadata collection

Each participant received a stool collection container with a lid and instructions for collecting the sample. When received by the research staff, a midsection of the stool sample was immediately stored in a 5 mL tube containing RNA*later* stabilising solution (Thermo Fisher Scientific) and frozen at −20 °C until arrival at UFPA, where samples underwent immediate DNA extraction.

Metadata collection for dietary information consisted of individual dietary habits interviews with urban-living participants and several interviews with ethnic leaders in the Native American populations. Further, stool samples were microscopically examined for intestinal parasites, and medical information regarding medication intake, previous and current diseases were obtained through the local medical staff responsible for each community or through individual interviews with urban-living participants.

### DNA extraction and 16S rDNA amplicon sequencing

Total DNA from faecal samples was extracted using the DNeasy PowerSoil Kit (Qiagen, Hilden, Germany) according to the manufacturer’s protocol with small modifications. Eluted DNA was quantified with fluorometry and subsequently stored at −20 °C. The next-generation sequencing library preparation was carried out according to the Illumina Metagenomic Sequencing Library Prep protocol with established primers and Illumina Nextera adapters targeting the V3–V4 region of the 16S rDNA, as follows: Bakt 341F (CCTACGGGNGGCWGCAG) and Bakt 805R (GACTACHVGGGTATCTAATCC). Libraries were subsequently pooled and quantified with TapeStation (Agilent, Santa Clara, CA) before being sequenced as 300 bp paired-end reads on the Illumina MiSeq platform in two sequencing runs, yielding an average 141,377 ± 52,840 raw reads per sample.

### Quality control and ASV classification

Initial raw data quality visualisation was carried out in FastQC^39^. All raw read quality control was performed in Quantitative Insights into Microbial Ecology (QIIME 2) software^40^. Reads were demultiplexed, denoised, merged, low-quality reads were trimmed, filtered and chimaeras were removed using DADA2^41^. After filtering, reads per sample were an average of 11,925 ± 4051. Amplicon sequence variant (ASV) data were generated by a Naive–Bayes machine-learning classifier in QIIME 2, which subsequently output a feature table identifying a total of 39,085 ASVs. A phylogenetic tree was inferred from ASVs using FastTree v.2.1.3^42^ and taxonomic classification was carried out in QIIME 2 using a Naive–Bayes classifier based on Greengenes database v.13.8 clustered at 97% similarity. Prior to taxonomic analyses, we removed one sample that yielded no mapped reads and any singleton ASVs and taxa with an unassigned phylum level.

### Data analyses

All alpha- and beta-diversity analyses were performed in R v.3.5.3^43^ using “phyloseq”^44^ and “vegan” packages^45^. Based on rarefaction curves (Supplemental information 1: Figure S12), we rarefied samples to 5000 reads to compute a number of observed species, Simpson, Chao1 and Shannon diversity metrics, which maintained 97.3% of samples and 75% of operational taxonomic units (OTUs) (Supplementary Data 1: Table 1). Statistical differences among population alpha diversity were tested using one-way ANOVA and Kruskal–Wallis *H* and pairwise comparisons were computed using *T* test and Wilcoxon’s rank-sum (Mann–Whitney *U*) test.

Beta diversity analyses were carried out by normalising unrarefied sample counts to relative abundances, removing taxa unobserved in at least 1% of samples and agglomerating taxa to genus level. We computed Bray–Curtis, Unifrac and Weighted Unifrac distances. Differences in dispersion from centroids in both Unifrac and Weighted Unifrac distances were tested with PERMANOVA. Pairwise PERMANOVA implemented in the *adonis* function of the “vegan”^45^ package in R was used to determine distances between population pairs. Spearman’s correlations were used to evaluate the relationship between alpha- and beta-diversity metrics. PCoA was computed in R using ggplot2 package^46^.

To compare our samples to data from other worldwide population gut microbiomes, we downloaded the filtered data from each study^4,5,7,17,22^ and performed OTU picking and taxonomic assignment with QIIME 2 using vsearch closed-reference based on Greengenes v.13.8 clustered at 97% similarity.

The most prevalent genus was determined considering taxa present in at least 1% of samples. The core microbiome taxa for each population were computed by determining taxa observed at least three times in the dataset and with a minimum frequency of 50% of samples within each population. Results were observed through an interaction network at genus and phylum levels created using the Cytoscape software (http://www.cytoscape.org/). Statistical analyses were carried out through hypergeometric enrichment tests to determine significant overlaps between core microbiomes of different populations using the *phyper* function in R v.3.5.3 “stats” package v.3.6.2^43^. *P* values were adjusted for multiple testing with the Benjamini–Hochberg method (Supplementary Data 1: Table 2).

Correlations between OTU abundances were calculated using SparCC software implemented in python^23^. For this analysis, we selected only the taxa present in at least 5% of samples to avoid any false correlations for low abundant taxa. Results were represented as a heatmap with Ward’s hierarchical clustering of taxa based on the dissimilarity matrix. The optimal number of clusters was determined based on *k*-means and PAM methods and validated with Hopkins statistic. An interaction network for visualisation was created with the Cytoscape software.

To test differences in taxa abundance among Amazonian and worldwide microbiome samples, we used ANCOM^24^, which accounts for the compositional nature of microbiome data. *W*-statistic cut-offs are reported in Supplementary Data 1: Table 3, but significance was defined as *W* > 0.9 in this analysis. Relative abundances are shown as the logarithm of re-scaled abundances as described by Hansen et al.^22^.

For functional prediction analyses, reads were taxonomically classified by closed-reference using vsearch with Greengenes v.13.5 database clustered at 97% similarity. KEGG pathways were predicted using PICRUSt 1 v.1.1.4^25^. Predicted metagenome functions were collapsed at level 2 pathways, and after removing eukaryote-related pathways, they were analysed using ANCOM^24^ to determine differentially abundant pathways among the population. Results were displayed in boxplots of relative abundances and *p* values were determined by Kruskal–Wallis *H* test and pairwise Wilcoxon’s rank-sum (Mann–Whitney *U*) test.

### Reporting summary

Further information on research design is available in the Nature Research Reporting Summary linked to this article.

## Supplementary information

Supplementary Information

Reporting Summary

Supplementary Data 1

## Data Availability

Datasets generated in this study are available in the European Nucleotide Archive repository under accession number: PRJEB39990. Worldwide data analysed in this research is deposited in EBI with IDs ERA387449 and ERP008799^4^, NCBI Sequence Read Archive with ID PRJNA395034^22^, European Nucleotide Archive with ID PRJEB30836^7^, QIIME database ID 1448^5^, and NCBI Sequence Read Archive ID PRJNA547608^17^.
