# Supplementary material for: The structure of Brazilian Amazonian gut microbiomes in the process of urbanisation
Source: NPJ Biofilms Microbiomes. 2021 Aug 5;7:65. doi: 10.1038/s41522-021-00237-0 (PMC8342711; doi:10.1038/s41522-021-00237-0)
Supplement: Supplementary file 1 — Supplementary Information [file 41522_2021_237_MOESM1_ESM.pdf]

Supplementary Data 1

Excel file containing seven data tables for 1) diversity indexes, 2) hypergeometric enrichment test results, 3) ANCOM results for genus level, 4) ANCOM results for family level, 5) ANCOM results for order, 6) PICRUSt pathway predictions, 7) ANCOM results for KEGG pathway predictions.

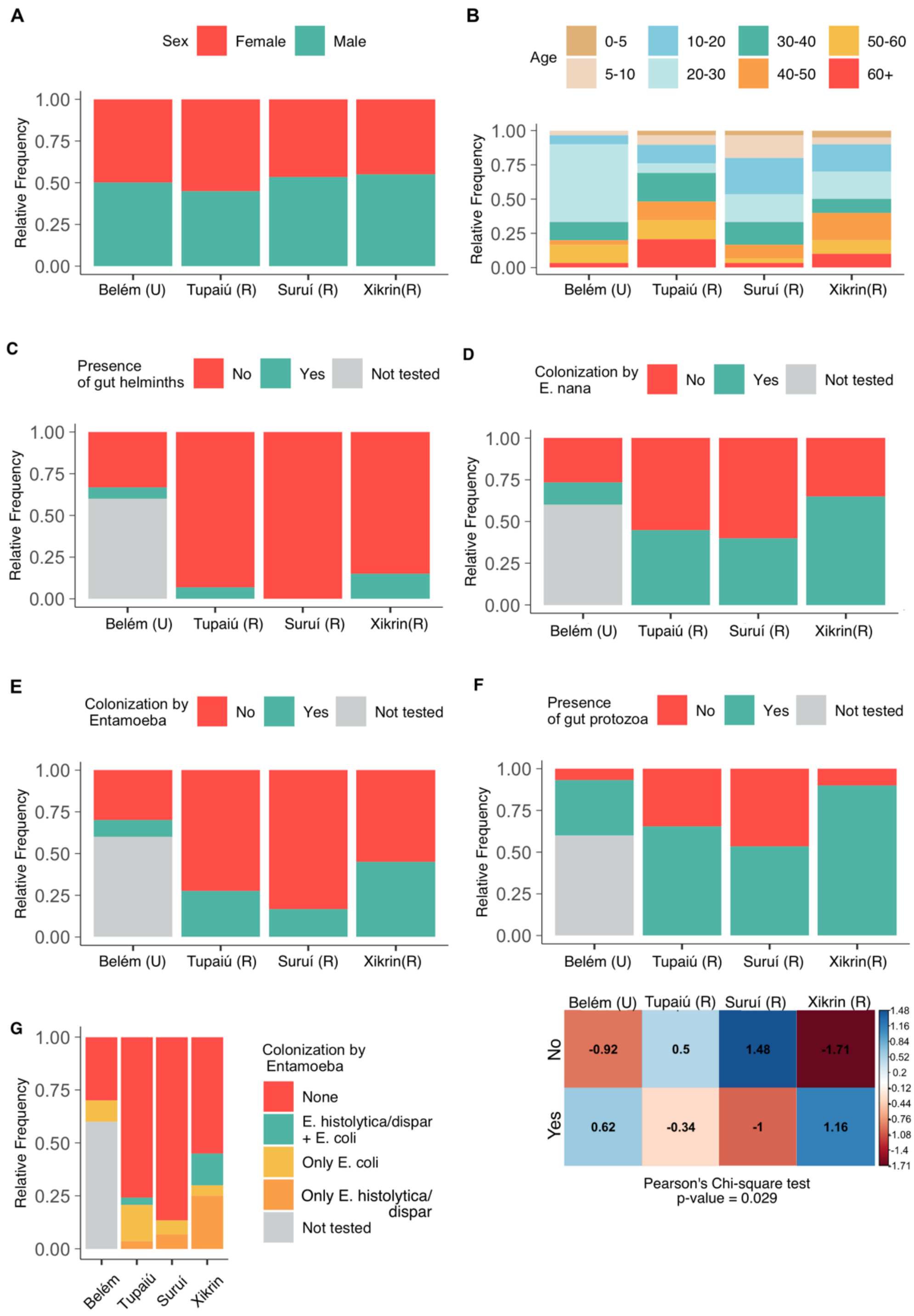

Figure S1

Metadata collection. A) Sex relative frequency per population. B) Relative frequencies of age groups per population. C) Presence of gut helminths as determined by microscopic examination of fecal samples. D) Gut colonisation by *Endolimax nana*. E) Gut colonisation by *Entamoeba* sp. F) Presence of gut protozoa as determined by microscopic examination of fecal samples (Pearson's Chi-Square test, p value = 0.029). G) Subtypes of *Entamoeba* sp. observed in fecal samples.

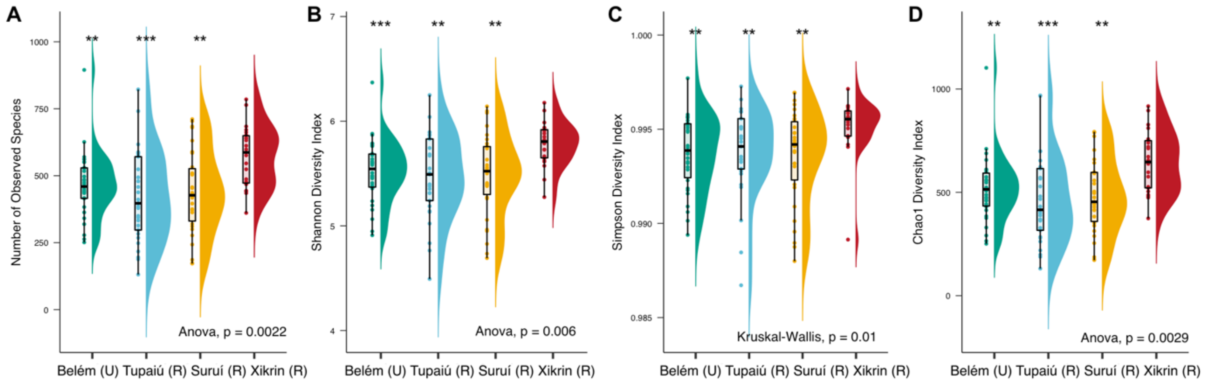

Figure S2

Alpha diversity measures. A) Number of observed species. B) Shannon diversity index. C) Simpson diversity index. D) Chao1 diversity index.

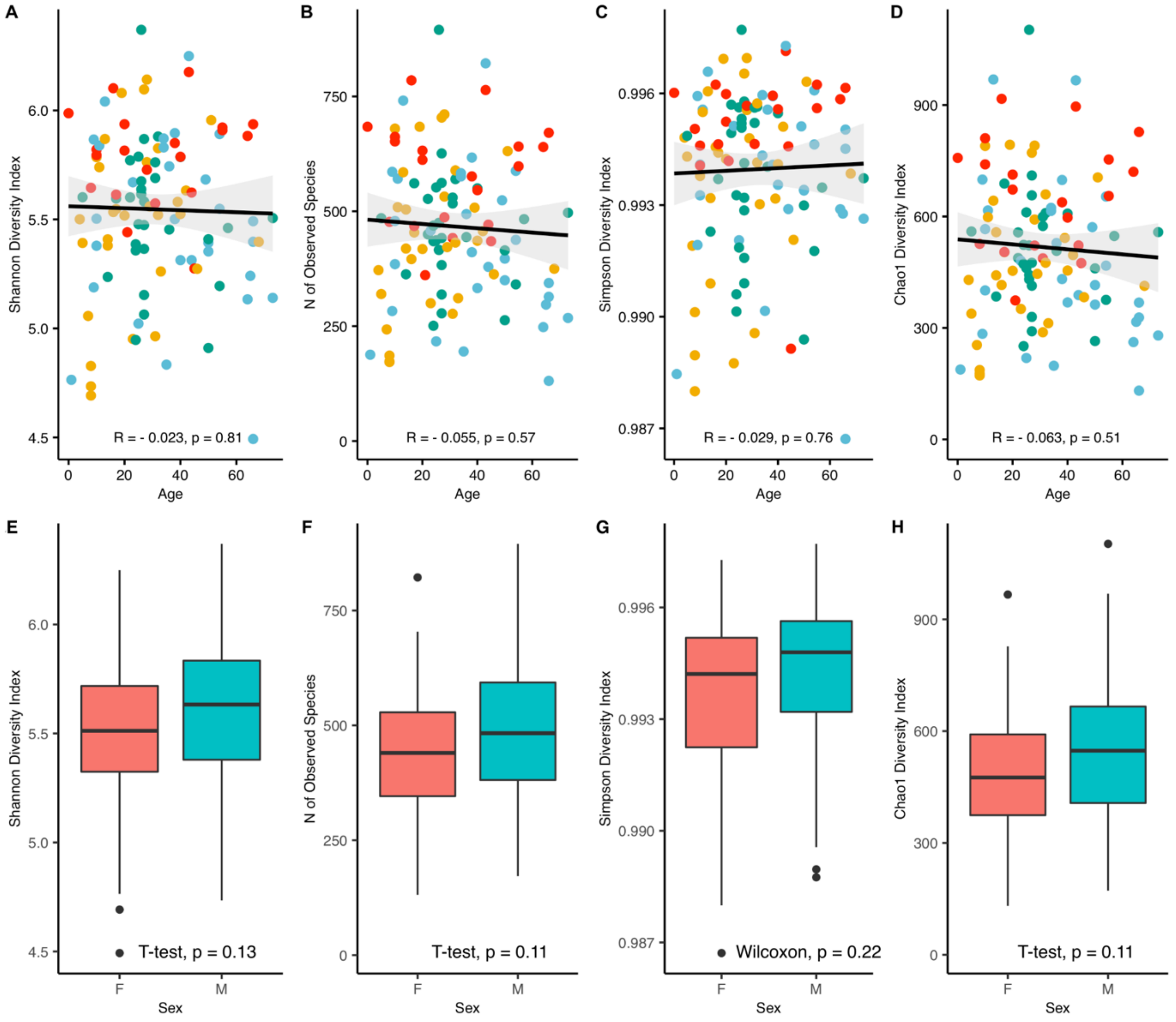

Figure S3

Box plots and scatter plots of alpha diversity indexes and information on participant age and sex. Significance was determined using Spearman correlation, t-test, and Wilcoxon rank sum (Mann Whitney U) tests. (Red = Xikrin (R), Yellow = Suruí (R), Blue = Tupaiú (R), Green = Belém (U)).

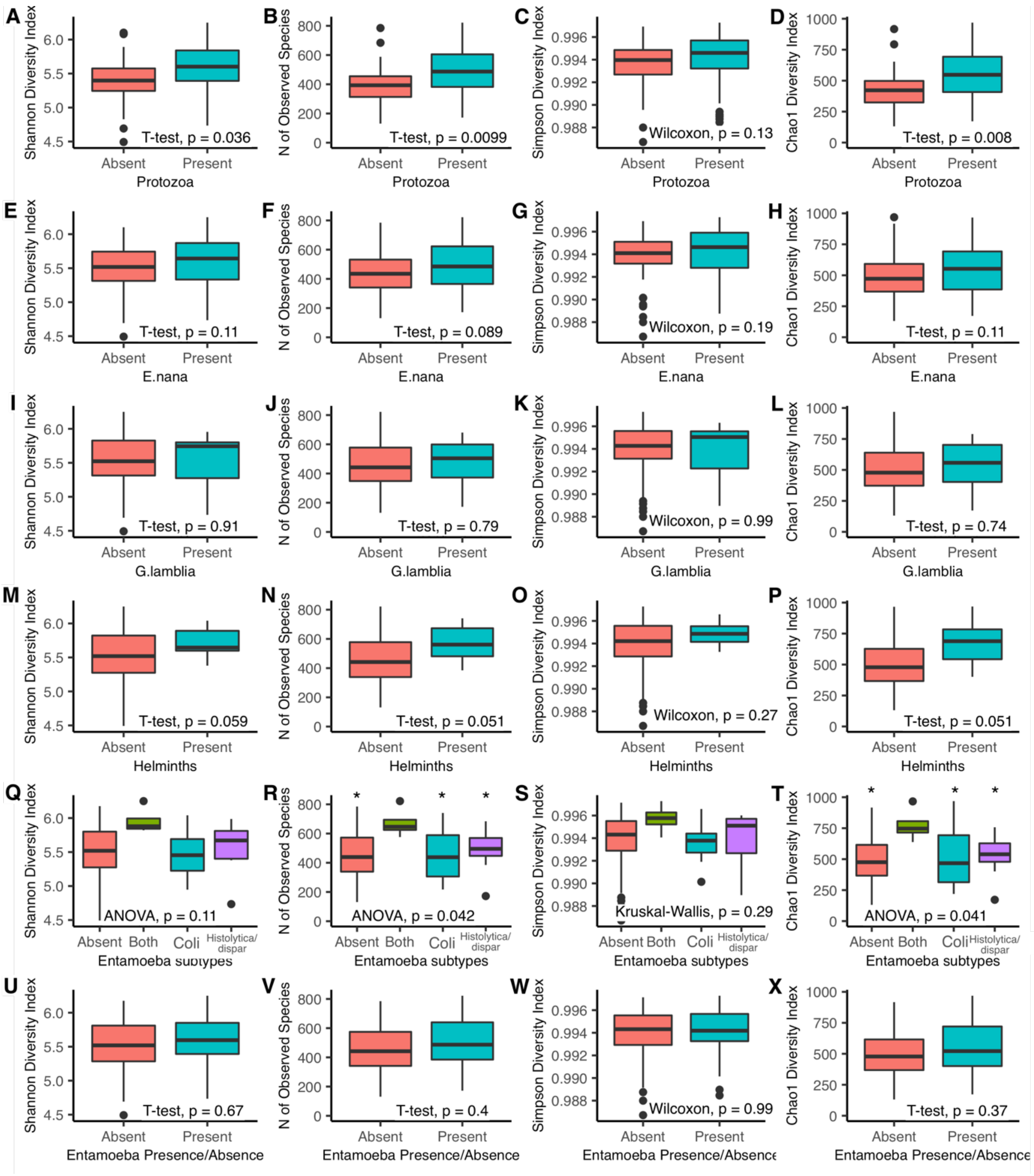

Figure S4

Box plots of alpha diversity indexes and metadata variables. A-D: Gut protozoa colonisation. E-H: Gut colonisation by *Endolimax nana*. I-L: Gut colonisation by *Giardia lamblia*. M-P: Gut helminth colonisation. Q-T: Gut colonisation by *Entamoeba* species. U-X: Gut colonisation by *Entamoeba* sp. Significance was determined using t-test, Wilcoxon rank sum (Mann Whitney U), ANOVA, and Kruskal-Wallis H tests.

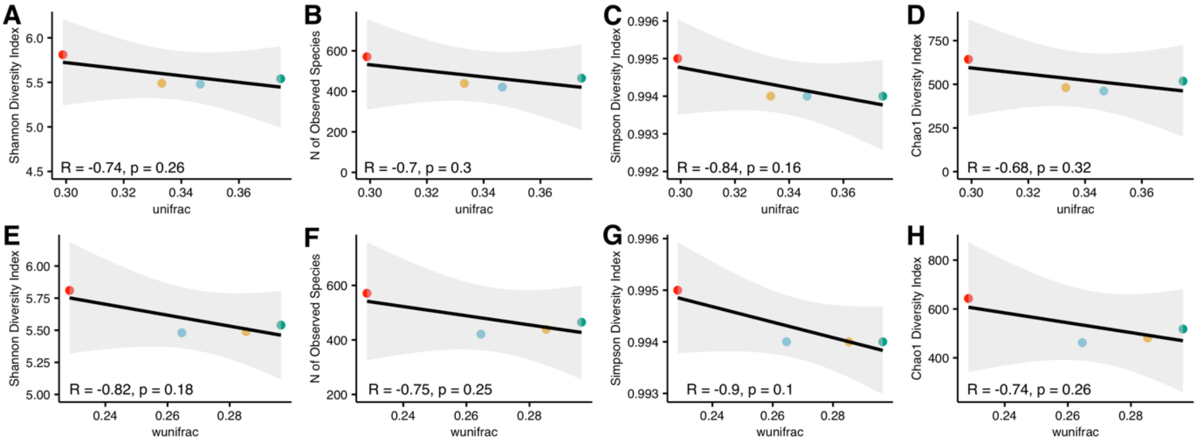

Figure S5

Scatter plots of within-population mean alpha diversity indexes versus weighted and unweighted Unifrac distances. Significance was determined using Spearman correlation tests. (Red = Xikrin (R), Yellow = Suruí (R), Blue = Tupaiú (R), Green = Belém (U)).

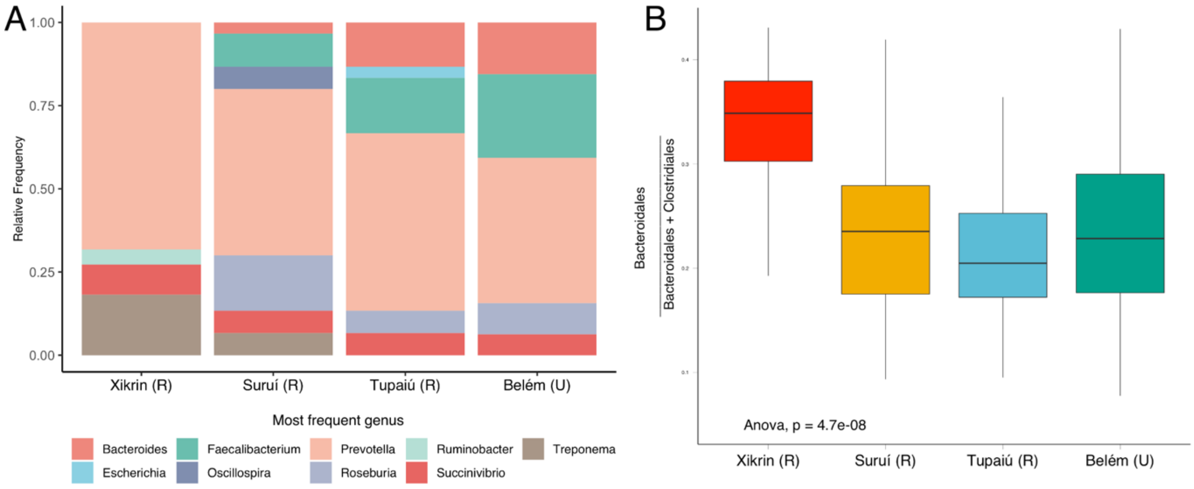

Figure S6

Taxa distribution among populations. A) Distribution of most prevalent genera among population groups. B) Relative proportion of *Bacteroidales* per total of *Bacteroidales* and *Clostridiales* across population groups.

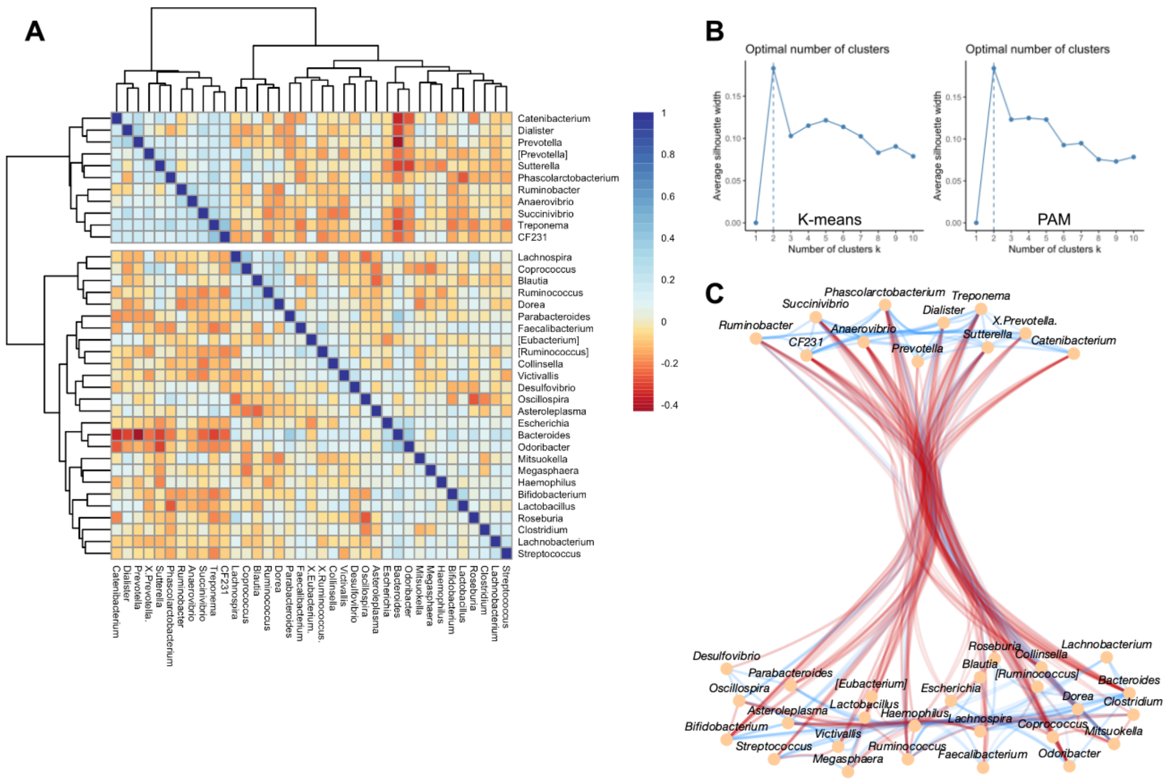

Figure S7

Microbial co-abundance analyses. A) Heatmap based on microbial sparse correlation coefficients. B) Co-abundance clustering tendencies based on k-means and PAM methods. C) Network representation of microbial co-abundance patterns, where blue edges indicate positive correlations and red represent negative correlations.

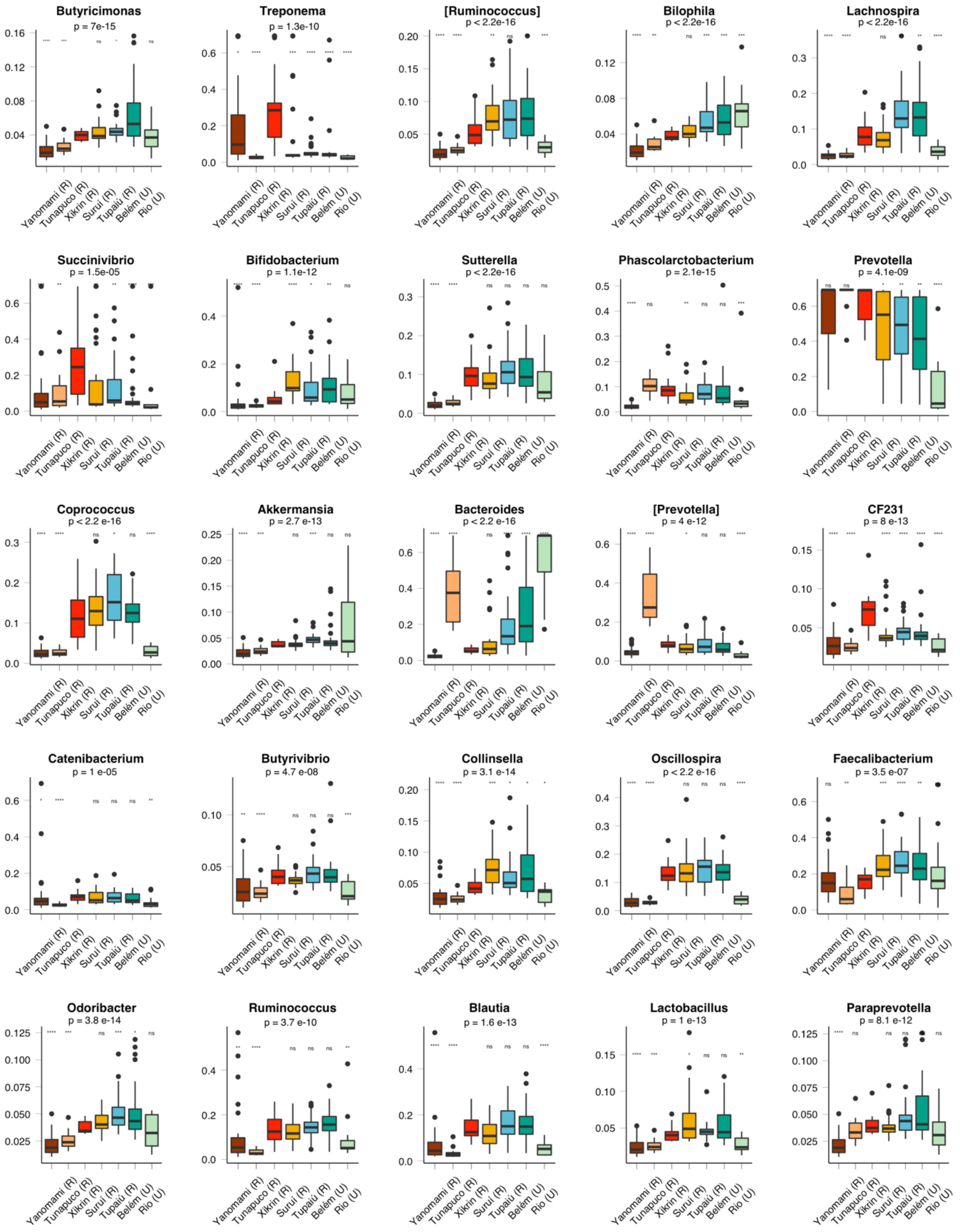

Figure S8

ANCOM results comparing differential abundances among taxa from other South American populations. Populations are ordered from most rural (left) to most urbanised (right) lifestyles. R = rural, SU = semi-urban, U = urban. Displayed *p*-values refer to Kruskal-Wallis H statistical tests.

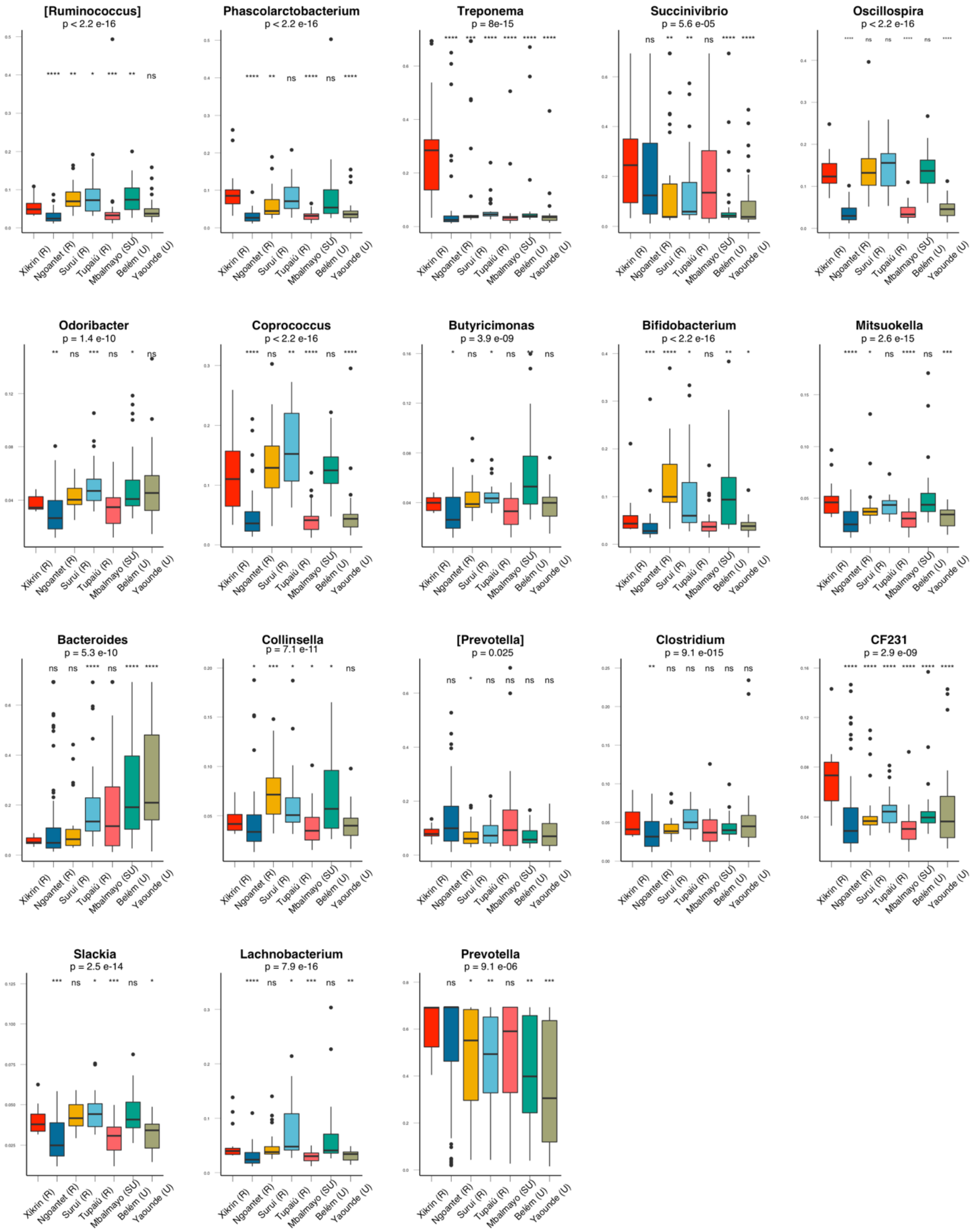

Figure S9

ANCOM results comparing differential abundances among taxa from African Cameroonian populations living in an urbanization gradient. Populations are ordered from most rural left) to most urbanized (right). R = rural, SU = semi-urban, U = urban. Displayed *p*-values refer to Kruskal-Wallis H statistical tests.

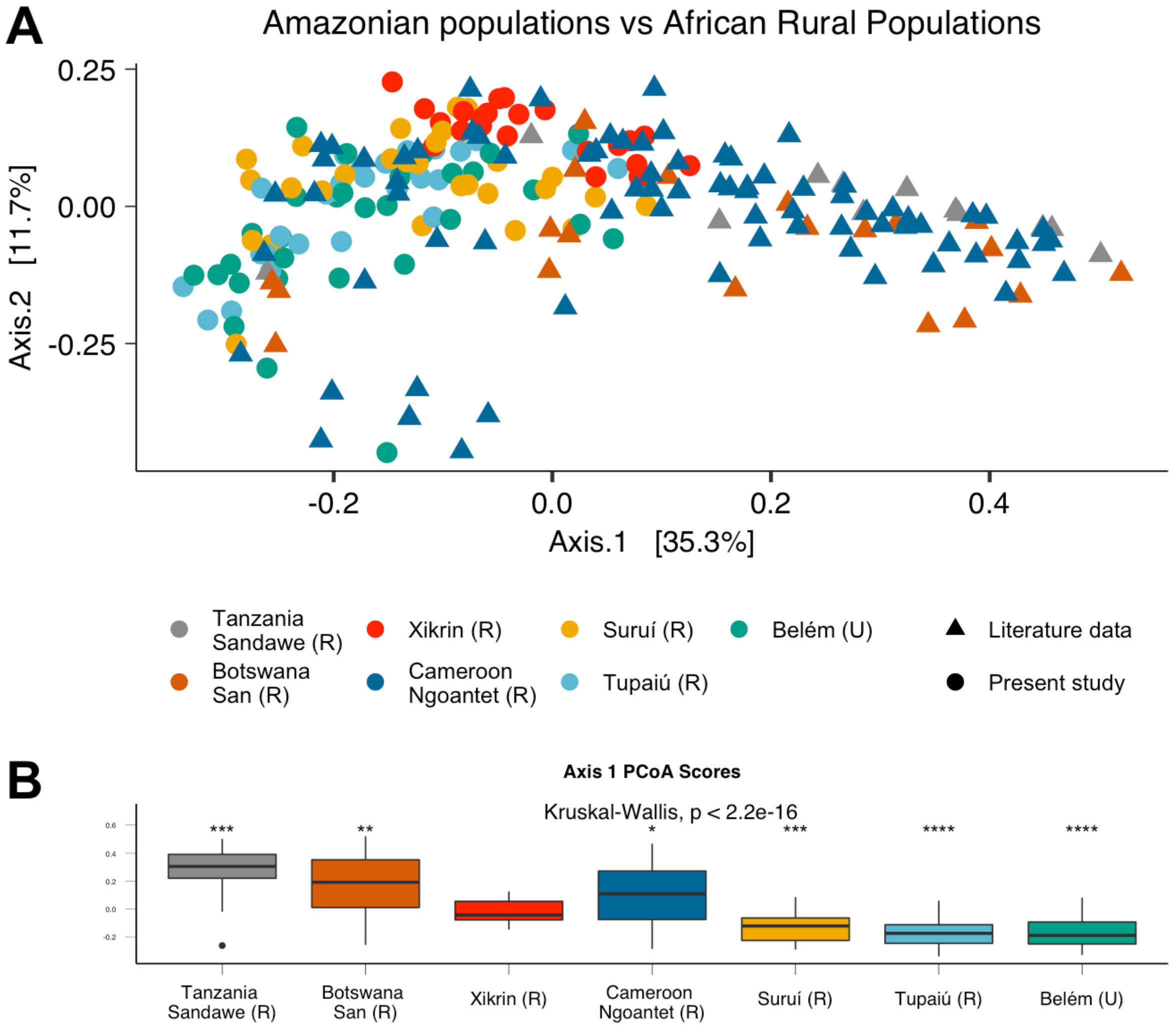

Figure S10

A) Principal Coordinate Analysis for visualizing Brazilian Amazonian samples compared to African individuals living in rural settings. B) Boxplots of PCA scores along axis 1. R = rural, SU = semi-urban, U = urban.

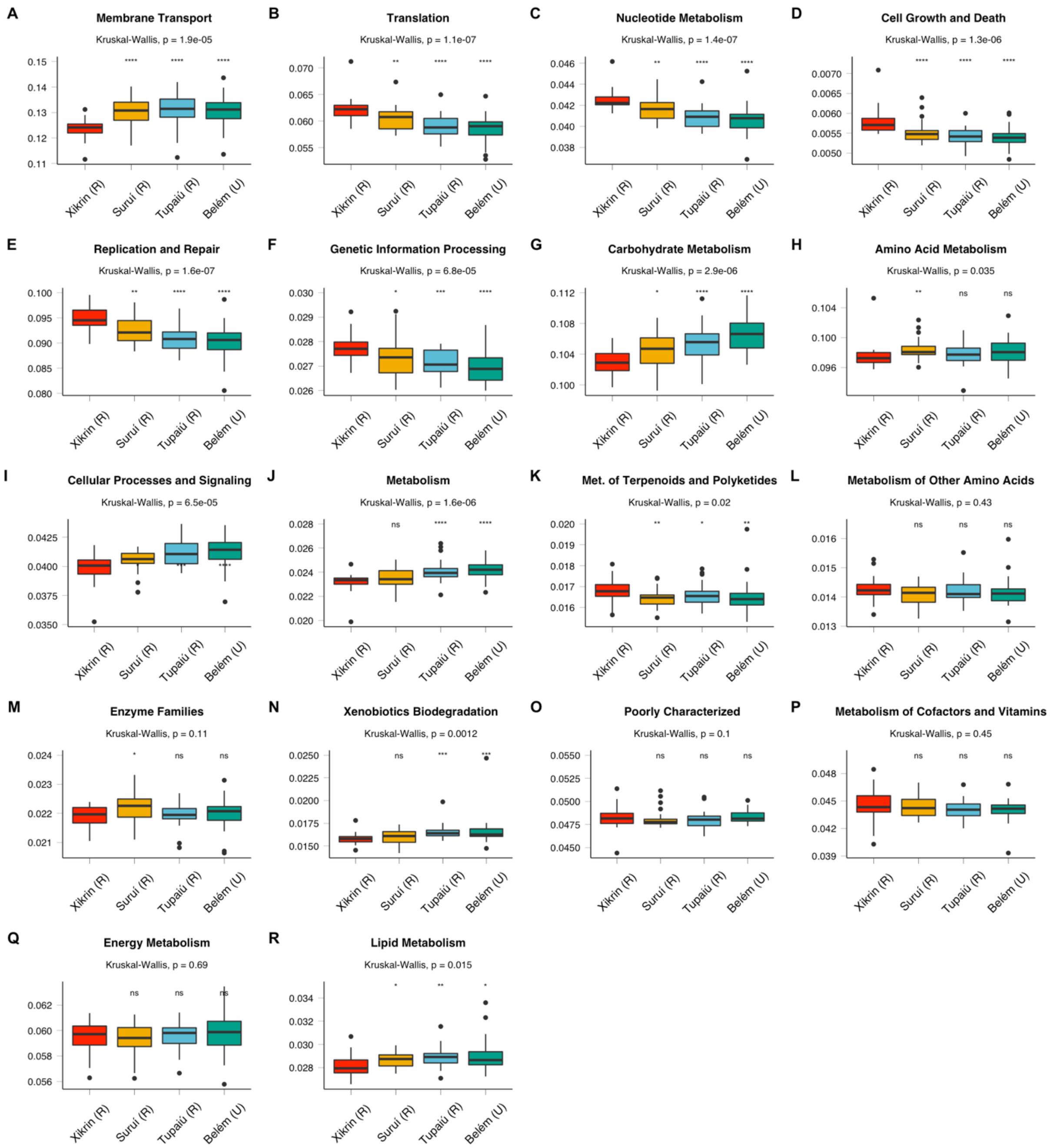

Figure S11

Boxplots of the relative abundance of differentially abundant KEGG Level 2 pathways as determined by ANCOM on PICRUSt functional prediction results. Here, significance is considered based on W = 0.6 ANCOM threshold. Global and pairwise statistical comparisons were carried out with Kruskal-Wallis H and Wilcoxon (Mann Whitney U) tests.

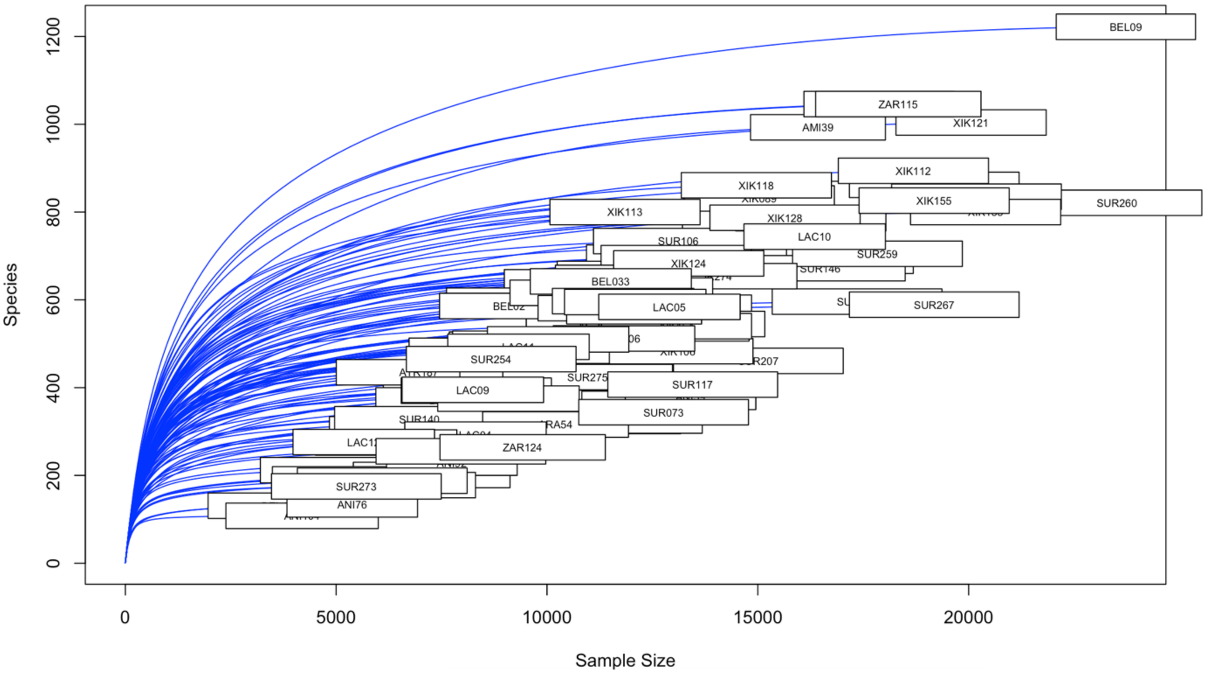

Figure S12

Species accumulation (rarefaction) curve to determine the number of species detected as a function of the number of sequences per sample. According to the rarefaction curve, the sampling effort was sufficient to describe the bacterial diversity in the samples.
