# Supplementary material for: The structure of Brazilian Amazonian gut microbiomes in the process of urbanisation
Source: NPJ Biofilms Microbiomes. 2021 Aug 5;7:65. doi: 10.1038/s41522-021-00237-0 (PMC8342711; doi:10.1038/s41522-021-00237-0)
Supplement: Supplementary file 2 — Reporting Summary [file 41522_2021_237_MOESM2_ESM.pdf]

nature research

Corresponding author(s): Ândrea Ribeiro-dos-Santos

Reporting Summary

Nature Research wishes to improve the reproducibility of the work that we publish. This form provides structure for consistency and transparency in reporting. For further information on Nature Research policies, see Authors & Referees and the Editorial Policy Checklist.

Statistical parameters

When statistical analyses are reported, confirm that the following items are present in the relevant location (e.g. figure legend, table legend, main text, or Methods section).

n/a	Confirmed	
☐	☒	The exact sample size (n) for each experimental group/condition, given as a discrete number and unit of measurement
☐	☒	An indication of whether measurements were taken from distinct samples or whether the same sample was measured repeatedly
☐	☒	The statistical test(s) used AND whether they are one- or two-sided<br>*Only common tests should be described solely by name; describe more complex techniques in the Methods section.*
☐	☒	A description of all covariates tested
☐	☒	A description of any assumptions or corrections, such as tests of normality and adjustment for multiple comparisons
☐	☒	A full description of the statistics including central tendency (e.g. means) or other basic estimates (e.g. regression coefficient) AND variation (e.g. standard deviation) or associated estimates of uncertainty (e.g. confidence intervals)
☐	☒	For null hypothesis testing, the test statistic (e.g. F, t, r) with confidence intervals, effect sizes, degrees of freedom and P value noted<br>*Give P values as exact values whenever suitable.*
☒	☐	For Bayesian analysis, information on the choice of priors and Markov chain Monte Carlo settings
☒	☐	For hierarchical and complex designs, identification of the appropriate level for tests and full reporting of outcomes
☒	☐	Estimates of effect sizes (e.g. Cohen's d, Pearson's r), indicating how they were calculated
☐	☒	Clearly defined error bars<br>*State explicitly what error bars represent (e.g. SD, SE, CI)*

Our web collection on statistics for biologists may be useful.

Software and code

Policy information about availability of computer code

Data collection	No software was used for sample collection. Deposited data that was analysed in this study was downloaded directly from each data repository.
Data analysis	Data analysis was carried out in Qiime 2 and R version 3.5.3.

For manuscripts utilizing custom algorithms or software that are central to the research but not yet described in published literature, software must be made available to editors/reviewers upon request. We strongly encourage code deposition in a community repository (e.g. GitHub). See the Nature Research guidelines for submitting code & software for further information.

Data

Policy information about availability of data

All manuscripts must include a data availability statement. This statement should provide the following information, where applicable:
- Accession codes, unique identifiers, or web links for publicly available datasets
- A list of figures that have associated raw data
- A description of any restrictions on data availability

Datasets generated in this study are available in the European Nucleotide Archive repository under accession number: PRJEB39990. Worldwide data analysed in this

research is deposited in EBI with IDs ERA387449 and ERP008799 4, NCBI Sequence Read Archive with ID PRJNA395034 22, European Nucleotide Archive with ID PRJEB30836 7, QIIME database ID 1448 5, and NCBI Sequence Read Archive ID PRJNA547608 17.

Field-specific reporting

Please select the best fit for your research. If you are not sure, read the appropriate sections before making your selection.

☒ Life sciences ☐ Behavioural & social sciences ☐ Ecological, evolutionary & environmental sciences

For a reference copy of the document with all sections, see nature.com/authors/policies/ReportingSummary-flat.pdf

Life sciences study design

All studies must disclose on these points even when the disclosure is negative.

Sample size	Sample sizes were determined based on literature data reporting adequate sample sizes for human metagenomics.
Data exclusions	We removed one sample from further analyses because it did not yield any reads in the sequencing run.
Replication	Data has been made available and technical procedures have been carefully described to allow adequate reproducibility of the data.
Randomization	We did not perform any experimental procedures with participants and this study aimed to solely characterise gut microbiome profiles in one-time stool samples.
Blinding	Investigator blinding to group allocation is not relevant to this study because this is not a randomized control or clinical trial.

Reporting for specific materials, systems and methods

Materials & experimental systems

n/a	Involved in the study	
☒	☐	Unique biological materials
☒	☐	Antibodies
☒	☐	Eukaryotic cell lines
☒	☐	Palaeontology
☒	☐	Animals and other organisms
☐	☒	Human research participants

Methods

n/a	Involved in the study	
☒	☐	ChIP-seq
☒	☐	Flow cytometry
☒	☐	MRI-based neuroimaging

Human research participants

Policy information about studies involving human research participants

Population characteristics	We sampled 114 individuals from four locations in the Brazilian Amazon including rural Native American communities (Xikrin, N = 22, Suruí, N = 30 and Tupaiú, N = 30), and one urban population (Belém, N = 32). Of these, 57 were males and 57 were females. Mean age was 30 years old.
Recruitment	Participants were recruited at random in each site.
